# Nuclear Vav3 is required for polycomb repression complex-1 activity in B-cell lymphoblastic leukemogenesis

**DOI:** 10.1038/s41467-022-30651-7

**Published:** 2022-06-01

**Authors:** R. C. Nayak, K. H. Chang, A. K. Singh, M. Kotliar, M. Desai, A. M. Wellendorf, M. Wunderlich, J. Bartram, B. Mizukawa, M. Cuadrado, P. Dexheimer, A. Barski, X. R. Bustelo, N. N. Nassar, J. A. Cancelas

**Affiliations:** 1 Hoxworth Blood Center, University of Cincinnati College of Medicine, Cincinnati, OH USA; 2 Division of Experimental Hematology and Cancer Biology, Cincinnati Children’s Hospital Medical Center, Department of Pediatrics, University of Cincinnati College of Medicine, Cincinnati, OH USA; 3 Department of Leukemia, Division of Cancer Medicine, The University of Texas MD Anderson Cancer Center, Houston, TX USA; 4 Epigenomics Data Analysis Core, Divisions of Allergy & Immunology and Human Genetics, Cincinnati Children’s Hospital Medical Center, Department of Pediatrics, University of Cincinnati College of Medicine, Cincinnati, OH USA; 5 Instituto de Biología Molecular y Celular del Cáncer, Centro de Investigación del Cáncer, Consejo Superior de Investigaciones Científicas (CSIC)-University of Salamanca, Salamanca, Spain; 6 Centro de Investigación Biomédica en Red de Cáncer (CIBERONC), Consejo Superior de Investigaciones Científicas (CSIC)-University of Salamanca, Salamanca, Spain; 7 Division of Biomedical Informatics, Cincinnati Children’s Hospital Medical Center, Department of Pediatrics, University of Cincinnati College of Medicine, Cincinnati, OH USA

**Keywords:** Acute lymphocytic leukaemia, Acute lymphocytic leukaemia

## Abstract

Acute B-cell lymphoblastic leukemia (B-ALL) results from oligo-clonal evolution of B-cell progenitors endowed with initiating and propagating leukemia properties. The activation of both the Rac guanine nucleotide exchange factor (Rac GEF) Vav3 and Rac GTPases is required for leukemogenesis mediated by the oncogenic fusion protein BCR-ABL. Vav3 expression becomes predominantly nuclear upon expression of BCR-ABL signature. In the nucleus, Vav3 interacts with BCR-ABL, Rac, and the polycomb repression complex (PRC) proteins Bmi1, Ring1b and Ezh2. The GEF activity of Vav3 is required for the proliferation, Bmi1-dependent B-cell progenitor self-renewal, nuclear Rac activation, protein interaction with Bmi1, mono-ubiquitination of H2A(K119) (H2AK119Ub) and repression of PRC-1 (PRC1) downstream target loci, of leukemic B-cell progenitors. Vav3 deficiency results in de-repression of negative regulators of cell proliferation and repression of oncogenic transcriptional factors. Mechanistically, we show that Vav3 prevents the Phlpp2-sensitive and Akt (S473)-dependent phosphorylation of Bmi1 on the regulatory residue S314 that, in turn, promotes the transcriptional factor reprogramming of leukemic B-cell progenitors. These results highlight the importance of non-canonical nuclear Rho GTPase signaling in leukemogenesis.

## Abstract (web summary)

Ph^+^ and Ph-like B-ALL remain poor prognosis leukemias. VAV3, a guanine nucleotide exchange factor, is activated and overexpressed in these leukemias. Here the authors reveal that leukemic VAV3 is predominantly nuclear. Nuclear VAV3, through its guanine nucleotide exchange factor and its effector nuclear RAC2, controls the repressive transcriptional activity of the polycomb repression complex-1 via nuclear AKT/PHLPP2 regulated BMI1.

## Introduction

The Philadelphia positive (Ph^+^) t(9;22) (q34;q11.2) translocation that generates the constitutively active BCR-ABL oncoprotein is frequently found in older adult (60%) and pediatric/adolescent-young adult (AYA) (25%) B-ALL cases^1,2^. The p190 fusion protein, the predominant form found in 60–80% of pediatric/AYA Ph^+^ B-ALL, a leukemia that derived from the transformation of a B-cell progenitor^3^. Ph-like B-ALL possess other driver mutations that induce the same transcriptional signatures as in Ph^+^ B-ALL^2^. Ph^+^ and Ph-like B-ALL have a much poorer prognosis compared to other cytogenetic or molecular abnormalities^4–7^. Genetic abnormalities including Bmi1 upregulation^8–10^, homozygous deletion of *INK4A/ARF*^11^ and mutations in the lymphoid-lineage transcription factor loci expressing *PAX5, IKZF1*, and *EBF1*^12–14^ collaborate with BCR-ABL in the progression to aggressive B-ALL. These mutations, however, are not present in all cases of children’s BCR-ABL^+^ B-ALL^13,15^ and, when present, they are usually monoallelic, which suggests that loss-of-heterozygosity drives B-ALL transformation. Recurrent epigenetic alterations in genes shown to be targets for aberrant DNA methylation have been identified in all B-ALL subtypes, suggesting that epigenetic, and not only genetic, modifications are required for leukemic transformation^16^. Our group has demonstrated that an oncogene-dependent epigenetic repression of genes involved in the proliferation and differentiation of B-cell progenitors plays a critical role in B-cell progenitor leukemogenesis^17^.

BMI1, a major component of the polycomb repression complex (PRC) type 1.4, is upregulated in patients with advanced stages of BCR-ABL-induced chronic myelogenous leukemia (CML)^8^ and through its repressive activity on the *Cdkn2a* locus^18^ and other less well-characterized activities results in leukemic transformation^19^. The overexpression of BMI1 reprograms lymphoid leukemia progenitors into a self-renewing and transplantable stem cell like phenotype via the repression of B-cell transcriptional programs and the induction of self-renewing genes^10,20,21^.

Rho GTPases play essential roles in transformation, initiation, and progression of BCR-ABL driven leukemias. Deficiency of Rac proteins impairs myeloid leukemogenesis induced by p210-BCR-ABL expression^22,23^. Vav proteins (Vav1, Vav2, and Vav3) are guanine nucleotide exchange factors (GEF) for Rac GTPases^24–29^. We have previously shown that genetic deletion of Vav3, but not Vav1 and Vav2, delays BCR-ABL-induced lymphoblastic leukemia and increases the therapeutic vulnerability during TKI treatments^30^. Human and murine BCR-ABL^+^ B-cell progenitors show increased expression and activation of Vav3^30^ and we have demonstrated that a first-in-class, Vav3 inhibitor significantly impairs human and murine B-cell progenitor lymphoblastic leukemogenesis in vitro and in vivo^31^. Vav3 deficiency impairs Rac GTPase activation, survival, and proliferation of BCR-ABL^+^ B-cell progenitors^30^. The survival of Vav3-deficient B-cell progenitors negatively correlates with the level of expression of pro-apoptotic proteins^30^. However, the underlying molecular mechanisms of Vav3 regulation of lymphoid leukemia B-cell progenitor proliferation remains unclear. In the present study, we demonstrate that the mechanism by which Vav3 controls leukemic cell proliferation is through a non-canonical nuclear function associated with the nucleation of PRC complex components and the control of PRC1.4 activity.

## Results

### Nuclear VAV3 predominantly controls proliferation of B-cell progenitors in a GEF-dependent manner

**Fig. 1:**
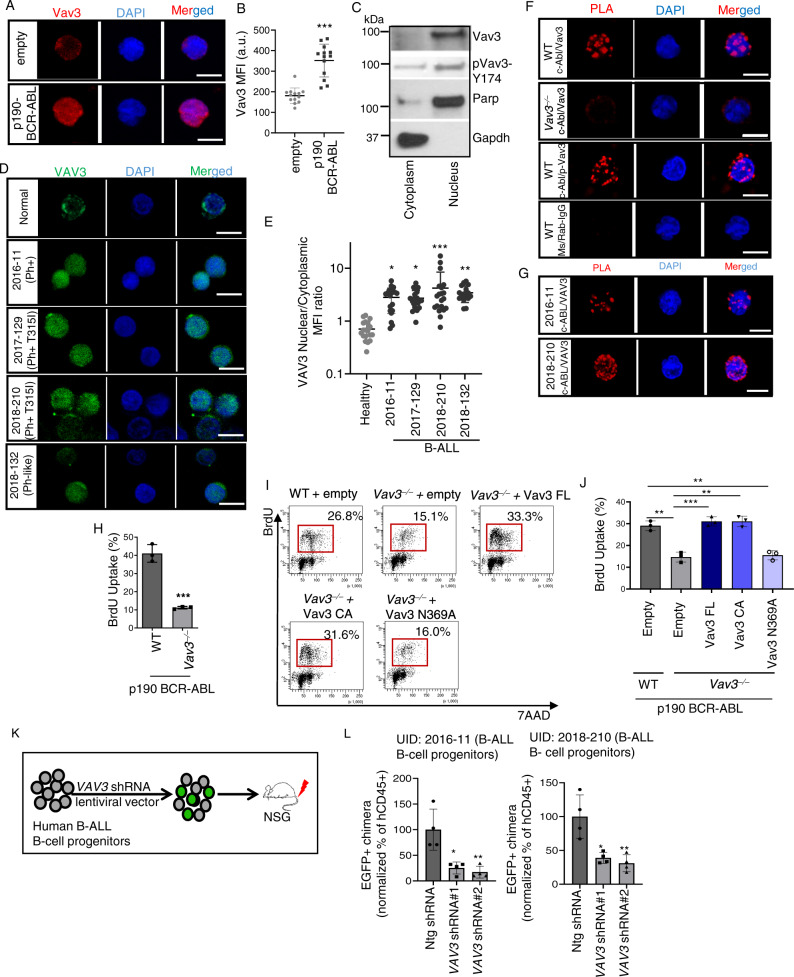
VAV3 expression is upregulated and predominantly nuclear in murine and human B-ALL B-cell progenitors, and its guanine nucleotide exchange activity is essential for leukemic B-cell progenitor proliferation. **A**, **B** Confocal immunofluorescence microscopic images (**A**) and quantification of mean fluorescence intensity (**B**) showing upregulation and nuclear distribution of Vav3 in p190-BCR-ABL^+^ murine B-cell progenitors (*n* = 13 per group). **C** Representative immunoblots for Vav3, pVav3-Y174, Gapdh and Parp in the cytoplasmic and nuclear fraction of p190-BCR-ABL^+^ B-cell progenitors. Vav3 is primarily distributed in the nuclear fraction. **D** Representative confocal immunofluorescence images of VAV3 expression in healthy donor and B-ALL patients derived B-cell progenitors (CD34^+^/CD19^+^). Mutation landscapes from top to bottom are: first row, normal karyotype; second row, BCR-ABL fusion; BCR/EXOSC2 fusion, CDKN2A loss; CDKN2B intron 1 truncation; third row, BCR-ABL (T315I); CD36 splice site 609+1G>A; SETD2 E282fs*19; SF3B1 T663I, sub; TLL2 G891fs*3; TP53 R248Q; fourth row, BCR-ABL (F359V; T315I); CDKN2A/B loss; IKZF1 loss; MLL2 S2173*; PAX5 Y129fs*64; fifth row, JAK1 L653 (subclonal) and R724H (subclonal); JAK2 R867Q; IGH/CRLF2; CDN2A loss exon 1; CDKN2B loss exon 2; FOXP1 R544*; ZRSR2 R448_R449insSRSR. **E** Nuclear/cytoplasm mean fluorescence intensity (MFI) ratio for VAV3 from primary normal and human B-ALL B-cell progenitors depicted in **D** (*n* = 16–20 per group). **F** Representative confocal images of PLA between c-Abl and Vav3 or p-Vav3-Y174 demonstrating physical proximity between BCR-ABL and Vav3 or p-Vav3. **G** Representative confocal images of PLA between c-ABL and VAV3 in Ph^+^ and Ph^+^ (T315I) patients B-cell progenitors. **H** Deficiency of Vav3 attenuates proliferation of leukemic B-cell progenitors as quantified by in vivo BrdU uptake assay (*n* = 3 per group). **I**, **J** Flow cytometry dot plots (**I**) and quantification (**J**) of proliferation (BrdU uptake) in Vav3-deficient B-cell progenitors ectopically expressing structure-function Vav3 mutants (FL- full-length, CA-constitutive active, Vav3 (N369A) (*n* = 3 per group). **K** Schematic diagram of human B-ALL model in NSG mice. **L** Normalized transduced human chimera (EGFP^+^) showing reduced chimera levels of VAV3 shRNA transduced Ph^+^ (BCR-ABL fusion; BCR/EXOSC2 fusion, CDKN2A loss; CDKN2B intron 1 truncation) and Ph^+^ (BCR-ABL (F359V; T315I); CDKN2A/B loss; IKZF1 loss; MLL2 S2173*; PAX5 Y129fs*64) transplanted groups in bone marrow (*n* = 4 per group). Engraftment of human leukemia (transduced and untransduced) was higher than 90% in all mice (see Supplementary Fig. 1F). Scale bar, 10 μm. Western blot and microscopic images are representative of a minimum of two independent experiments. Data are plotted as mean ± SD in a minimum of two independent experiments. Statistical significance was determined using the unpaired Student-t or Anova tests when more than two groups were compared. *p <  0.05; **p <  0.01; ***p <  0.001.

Previous results have shown that VAV3 activity plays important tumorigenic functions in a number of cancer models^32–35^. Our previous work has also shown that Vav3 plays roles in acute lymphoblastic leukemia, a function linked to the regulation of the survival of B-cell progenitors^30^. This work, however, could not identify the signaling mechanism involved in this process^30^. It was inferred that this leukemogenic process was associated with the engagement of the canonical GEF activity of Vav3 at the plasma membrane. To approach this issue, we first examined the expression and cellular distribution of this GEF in murine and human BCR-ABL^+^ B-ALL cell progenitors. Consistent with our earlier study^30^, we found that p190-BCR-ABL induces Vav3 expression with predominant nuclear localization in murine B-cell progenitors (Fig. 1A, B). Western blot analyses using different subcellular cell extracts revealed that Vav3 is preferentially located in the nucleus under these conditions (Fig. 1C). Nuclear Vav3 is probably in an active state, as inferred by the detection of substantial levels of phosphorylation of the key residue involved in the activation step of the GEF (Y174) (Fig. 1C). Similarly, human B-cell progenitors from Ph^+^ or Ph-like B-ALL patients, containing secondary pathogenic mutations (including loss of CDKN2A/B, mutant PAX5 and loss of IKZF1; see Supplemental Methods), show increased VAV3 expression with a predominant nuclear distribution (Fig. 1D, E).

As expected^36^, a significant fraction of BCR-ABL localizes in the nucleus of murine and human B-ALL B-cell progenitors (Supplementary Fig. 1A). To determine whether ABL and VAV3 interact, we performed a proximity ligation assay (PLA) that allows the in situ detection of the protein-protein interactions with subcellular location resolution^37^. Using this method we found that the nuclear fraction of VAV3 does reside in close proximity with BCR-ABL in both murine and human B-ALL cell progenitors (Fig. 1F, G). By contrast, no interaction is seen in B-cell progenitors from non-leukemic mice (Supplementary Fig. 1B). These data indicate that BCR-ABL and VAV3 are in close physical proximity in transformed B cell progenitors.

The nuclear distribution of leukemic VAV3 prompted us to investigate its role in regulating genes involved in leukemic B-lymphoid progenitor proliferation using a *Vav3*^−/−^ cell model (Supplementary Fig. 1C). We found that the Vav3 deficiency significantly attenuates the proliferation of p190-BCR-ABL^+^ B-cell progenitors (Fig. 1H). Re-expression of either full length (FL) Vav3 or constitutively active (CA) Vav3 rescues the proliferation of *Vav3*^−/−^ leukemic progenitors (Fig. 1I, J and Supplementary Fig. 1D, E). The expression of a catalytically inactive version of Vav3 (N369A, referred to as Vav3 NA) does not show this rescue activity (Fig. 1I, J and Supplementary Fig. 1D, E), indicating that this biological activity is GEF- and GTPase-dependent. Likewise, we found that xenotranplants of shRNA-mediated knockdown of *VAV3*^30^ in leukemic progenitors from a Ph^+^ B-ALL patient (CD34^+^/CD19^+^; Fig. 1K) reduces by ~60–75% the level of human leukemic progenitor chimera (hCD45^+^ hCD34^+^ hCD19^+^ EGFP^+^) present in the bone marrow of the transplanted immunodeficient recipients (Fig. 1L and Supplementary Fig. 1F). Taken together, these data indicate that VAV3 has a crucial role in the proliferation of Ph^+^ B-ALL progenitors.

### Nuclear Vav3 interacts with Bmi1 and regulates PRC1 activity

**Fig. 2:**
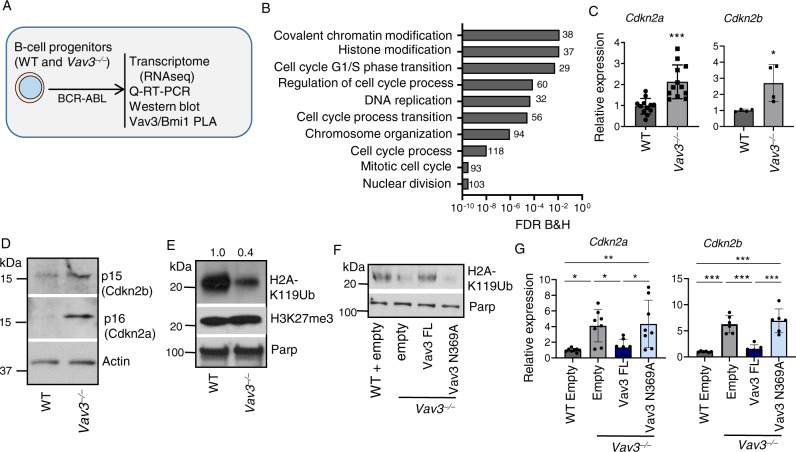
Nuclear Vav3 regulates Cdkn2a and Cdkn2b expression by modulating PRC1 activity. **A** Schema depicting the assays performed in Fig. 2. **B** Comparative transcriptome and gene-ontology [molecular and biological functions] of differentially expressed genes in WT and *Vav3*^*−*/*−*^ leukemic B-cell progenitors showing the differential regulation of genes involved in nuclear division, cell cycle regulation, G1/S phase transition, and covalent modifications of histone. **C** Quantitative real time PCR (Q-RT-PCR) analyses of *Cdkn2a* and *Cdkn2b* in *Vav3* deficient p190-BCR-ABL^+^ B-cell progenitors in comparison to their WT counterparts (*n* = 4–12 per group). **D** Representative immunoblots for p16/Ink4A, p15/Ink4b and Actin in the whole cell lysates of WT and *Vav3*^*−*/*−*^ leukemic B-cell progenitors. **E** Representative immunoblots for H2AK119Ub, H3K27me3 and Parp in the nuclear fraction of WT and *Vav3*^*−*/*−*^ leukemic B-cell progenitors. **F** Representative immunoblots for H2AK119Ub and Parp in the nuclear fraction of WT leukemic B-cell progenitors transduced with empty vector and *Vav3*^*−*/*−*^ leukemic B-cell progenitors transduced with empty vector or Vav3 FL or Vav3N369A GEF mutant vectors. **G** Q-RT-PCR analyses of *Cdkn2a* and *Cdkn2b* in empty vector transduced WT and empty or Vav3 or Vav3N369A vector transduced *Vav3*^*−*/*−*^ leukemic B-cell progenitors (*n* = 6–8 per group). Data are presented as mean ± SD of a 2 or 3 independent experiments. Statistical significance was determined using the unpaired Student-*t* or Anova tests when more than two groups were compared. *p < 0.05; **p < 0.01; *p* < 0.001.

To unravel the mechanism involved in the Vav3-mediated regulation of p190-BCR-ABL^+^ leukemogenesis, we first performed genome-wide expression analyses in Vav3-deficient and control transformed B cell progenitors (Fig. 2A). We identified the differential expression of 1110 genes (450 upregulated and 660 downregulated) in *Vav3*^−/−^ leukemic B-cell progenitors (<0.5 to >1.5-fold, *p* <  0.05; Supplementary Data 1). Gene ontology (GO) analyses revealed the differential expression of genes involved in nuclear division, regulation of cell cycle, G_1_/S phase transition, chromosome organization, and covalent histone modifications (Fig. 2B and Supplementary data 2). We also found similarities of this transcriptome with those previously identified in cells bearing gain-of-function of the cyclin-dependent kinase inhibitors *Cdkn2a* and *Cdkn2b* (GSE125841)^38^ and of the B-cell differentiation master regulator *Pax5* (GSE126375)^39^. All those genes are frequently altered in B-ALL^40,41^. Consistent with this, we found that the deficiency of Vav3 results in the upregulation of all those transcripts (Fig. 2C and Supplementary Fig. 2A) as well as in increased expression of both p16Ink4a and p15Ink4b (Fig. 2D). However, the expression of *Ebf1* and *Ikzf1*, two other crucial B-cell differentiation factors, remains unchanged in the absence of Vav3 (Supplementary Fig. 2A).

Transcriptional profiles of human BCR-ABL^+^ lymphoid and myeloid blast crisis progenitors are enriched for PRC1- and depleted for PRC2-related gene sets, respectively^42^. Our transcriptional data also suggest a role for Vav3 in the regulation of histone covalent modifications like those produced by the PRC complexes (Fig. 2B and Supplementary Data 2). This led us to analyze the status of PRC activity in *Vav3*^−/−^ leukemic progenitors. We found that the loss of Vav3 decreases (60 ± 1%, *p* < 0.05) the global level of H2A(K119) mono-ubiquitination (H2AK119Ub), a hallmark of PRC1 activity (Fig. 2E). However, the Vav3 deficiency does not affect the levels of PRC2 activity as assessed by the analysis of the global levels of H3K27 tri-methylation (H3K27me3) (Fig. 2E). The re-expression of full-length Vav3 restores global H2AK119Ub levels and transcriptional repression of *Cdkn2a/2b* (Fig. 2F, G). Further underscoring the GEF-dependency of this process, we could not observed any rescue activity when using the catalytically inactive version of Vav3 (Fig. 2F, G).

**Fig. 3:**
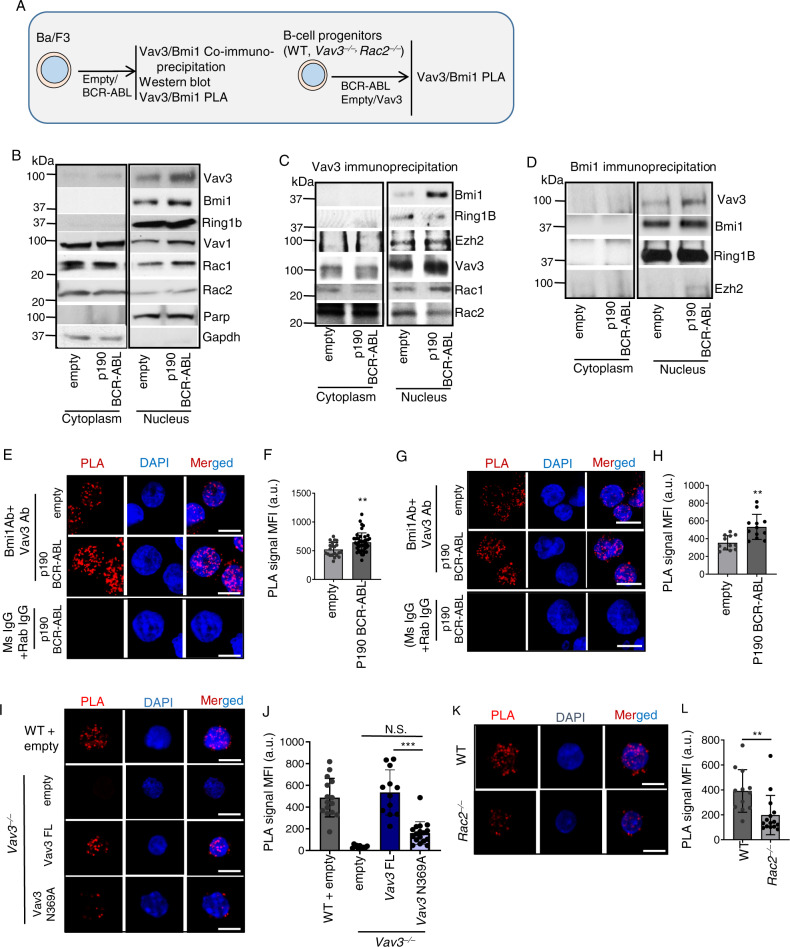
Nuclear Vav3 interacts with components of PRC1 complex. **A** Schema depicting the assays performed in Fig. 3. **B**–**D** Western blot analyses of input (**B**), α−Vav3 Ab immunoprecipitated (**C**), and α−Bmi1 Ab Immunoprecipitated (**D**) cytoplasmic and nuclear fractions of empty vector and p190-BCR-ABL transduced Ba/F3 cells. **E** Confocal microscopic images of PLA between Vav3 and Bmi1 in empty vector or p190-BCR-ABL transduced Ba/F3 cells. **F** Quantification of the mean fluorescence intensity of the PLA signals depicted in **D** (*n* = 16–43 per group). **G** Confocal microscopic images of PLA between Vav3 and Bmi1 in empty vector or p190-BCR-ABL transduced murine B-cell progenitors. **H** Quantification of the mean fluorescence intensity of the PLA signals depicted in **G** (*n* = 12 per group). Nuclear Vav3 and Bmi1 reside in close proximity and p190-BCR-ABL expression enhances PLA signal. **I**, **J** Confocal microscopic images of PLA between Vav3 and Bmi1 (**I**) and quantification (**J**) in empty vector transduced WT leukemic murine B-cell progenitors and empty vector/Vav3 FL/Vav3 GEF inactivating mutant lentiviral vector transduced *Vav3*^−/−^- leukemic murine B-cell progenitors (*n* = 11–17 per group). **K**, **L** Confocal microscopic images of PLA between Vav3 and Bmi1 (**K**) and quantification (**L**) in WT and *Rac2*^−/−^ leukemic murine B-cell progenitors (*n* = 12–15 per group). Scale bar, 10 μm. Data are presented as mean ± SD of a 2 or 3 independent experiments. Statistical significance was determined using the unpaired Student-*t* or Anova test when more than two groups were compared. Differences in survival were examined using the log-rank *P* test. *p <  0.05; **p <  0.01; ***p <  0.001.

The interaction between the enzyme Ezh2, a known member of the PRC2 complex, and Vav family proteins has been reported before^43^. The dynamic interaction between PRC2 and PRC1 has also been described^44^. Based on our data, we hypothesized that VAV3 and BMI1, a crucial component of the PRC1 complex protein responsible for H2AK119 mono-ubiquitination^45^, could be involved in this process. To test this idea, we performed Bmi1 and Vav3 co-immunoprecipitations using both the cytoplasmic and nuclear extracts from Ba/F3 cells (Fig. 3A). Similar to primary murine B-cell progenitors, we observed that p190-BCR-ABL promotes the expression and nuclear localization of Vav3 in these cells (Fig. 3B and Supplementary Fig. 2B, C). As expected, the PRC proteins Bmi1 and Ring1B are present in the nuclear fraction (Fig. 3B). We found that Vav3 immunoprecipitated with Bmi1, Ring1B, and the PRC2 protein Ezh2 (Fig. 3C). Conversely, we found that Vav3 also co-immunoprecipitates with Bmi1 in these cells (Fig. 3D). Although predominantly cytoplasmic, we also found that the Rac1 and Rac2 GTPases are co-immunoprecipitated with nuclear Vav3 in p190-BCR-ABL-expressing Ba/F3 cells (Fig. 3B, C).

Validating these observations, we also found that Vav3 and Bmi1 are in close proximity within the nucleus (Fig. 3E, F). Bmi1 and Vav3 also associate with primary murine progenitors, and interaction is boosted by the ectopic expression of p190-BCR-ABL (Fig. 3G, H). Compared to the full-length Vav3 protein, the interaction of the catalytically deficient Vav3(N369A) mutant proteins with Bmi1 is reduced ~2-fold (Fig. 3I, J). A similar effect is seen in Rac2-deficient leukemic B-cell progenitors (Fig. 3K, L), further indicating that the Vav3–Bmi1 interaction that takes place inside the nucleus is both GEF and GTPase-dependent.

### Vav3 collaborates with Bmi1 in B-cell leukemogenesis

**Fig. 4:**
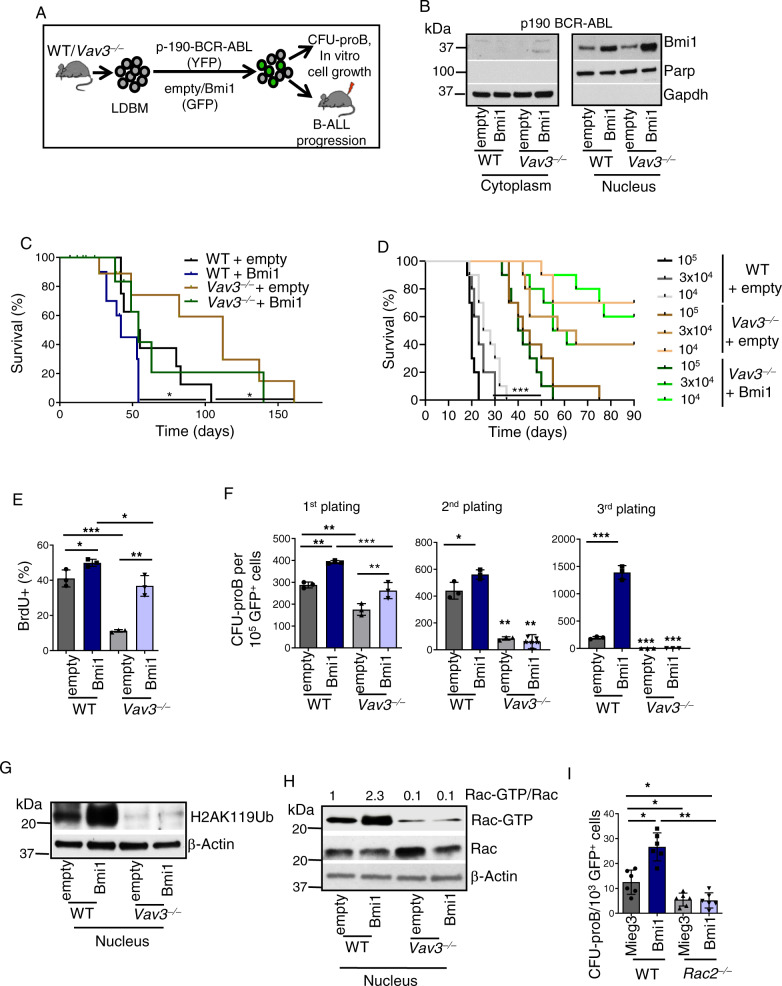
Deficiency of Vav3 abrogates oncogenic effect of Bmi1 over-expression in leukemic B-cell progenitors. **A** Wild type or *Vav3*^−/−^ LDBM cells co-transduced with p190-BCR-ABL retroviruses and empty or Bmi1 lentiviruses were transplanted into lethally irradiated C57Bl/10 mouse for the development of p190-BCR-ABL-induced B-cell acute lymphoblastic leukemia (B-ALL). **B** Representative Immunoblots for Bmi1, Parp and Gapdh in the cytoplasmic and nuclear extracts of B-cell progenitors derived from p190-BCR-ABL retrovirus and empty or Bmi1 lentivirus co-transduced and transplanted leukemic mice (*n* = 10 mice per group). **C** Kaplan–Meier overall survival analyses of primary recipient mice transplanted with WT or *Vav3*^*−*/*−*^ LDBM cells (10^6^ cells/mouse) co-transduced with p190-BCR-ABL retroviruses and empty or Bmi1 lentiviruses. Vav3 deficiency resulted in significant delay in chimeric mouse death. Bmi1 overexpression in WT, but not of *Vav3*^*−*/*−*^, leukemic cells resulted in significantly decreased latency to chimeric mouse death. **D** Kaplan–Meier survival analyses (90-days) of secondary recipient mice transplanted with 10^4^ (dotted lines, black-WT empty; gray-*Vav3*^−/−^ empty; light blue-*Vav3*^−/−^ Bmi1), 3 × 10^4^ (dashed lines, black-WT empty; gray-*Vav3*^−/−^ empty; light blue-*Vav3*^−/−^ Bmi1), and 10^5^ (solid lines, black-WT empty; gray-*Vav3*^−/−^ empty; light blue-*Vav3*^−/−^ Bmi1) leukemic B-cell progenitors derived from primary leukemic mice. Vav3 deficiency significantly prolongs the survival. No significant difference in survival between *Vav3*^*−*/*−*^ + empty and *Vav3*^*−*/*−*^ + Bmi1 at any of the three cell doses tested was found (*n* = 10 mice per group). **E** Quantification of BrdU uptake of WT and *Vav3*^−/−^ B-cell progenitors co-expressing p190-BCR-ABL and Bmi1. Vav3 deficiency partially impairs the Bmi1 overexpression effect (*n* = 3 per group). **F** Serial plating of CFU-proB showing abrogation of CFU generating ability of Vav3 deficient empty or Bmi1-transduced p190-BCR-ABL expressing B cell progenitors. **G** Representative immunoblots of H2AK119Ub, and β-actin in empty or Bmi1 over-expressed WT or *Vav3*^*−*/*−*^ leukemic B-cell progenitors (*n* = 3 per group). **H** Rac activation assay in empty or Bmi1 over-expressed WT or *Vav3*^*−*/*−*^ leukemic B-cell progenitors. **I** CFU-proB content of empty or Bmi1 over expressing WT or *Rac2*^*−*/*−*^ leukemic B-cell progenitors (*n* = 6 per group). Data are presented as mean ± SD in two or three independent experiments. Statistical significance was determined using the unpaired Student-t or Anova tests when more than 2 groups were compared. *p < 0.05; **p <  0.01; ***p < 0.001.

To determine whether Vav3 and Bmi1 interaction modulates the PRC1 activity and p190-BCR-ABL-induced B-ALL progression, we co-transduced WT and *Vav3*^*−*/*−*^ low-density bone marrow cells (LDBM) with p190-BCR-ABL (plus EYFP) and Bmi1 (plus EGFP) bicistronic integrating vectors, and then transplanted them into lethally irradiated congenic female mice to monitor leukemogenesis (Fig. 4a). As control, we used vectors expressing EGFP alone. We confirmed nuclear Bmi1 overexpression in the Bmi1-transduced leukemic progenitors (FSC^hi^ EYFP^+^EGFP^+^B220^lo^CD19^+^IgM^–^CD43^+^) (Supplementary Fig. 3 and Fig. 4b). Consistent with our previous reports^10,30^, the transplantation of p190-BCR-ABL-expressnig WT or *Vav3*^−/−^ leukemic LDBM cells leads in both cases to leukemogenesis as demonstrated by the detection of lymphadenopathies, hepatosplenomegaly, and extraosseus tumors in head and neck (data not shown). However, this process is significantly delayed in mice recipient of Vav3-deficient LDBM cells (Fig. 4C). The overexpression of Bmi1 in WT leukemic cells results in the development of a more aggressive leukemia that results in ~40% of the transplanted mice dying much faster than controls (Fig. 4C). However, such an acceleration in in vivo leukemogenesis in not observed when Bmi is ectopically expressed in Vav3-deficient cells (Fig. 4C). This differential behavior was further amplified when using serial transplantations of different cell doses of B-cell progenitors derived from WT and *Vav3*^−/−^ primary leukemic mice (Fig. 4D). Whole exome sequencing and copy number variation analyses identified no large-scale mutations or copy number variation in regions containing genes associated with leukemogenesis in the serially propagated WT and *Vav3*^−/−^ leukemias (Supplementary Data 3). However, we did find a heterozygous point mutation in Pax5 (P80R) in three out of the four analyzed secondary WT leukemias but not in the *Vav3*^−/−^ leukemias. Interestingly, this difference in survival of secondary recipients induced by the Vav3 deficiency is not rescued by Bmi1 overexpression (Fig. 4D), reinforcing the concept that Vav3 is required for Bmi1-induced leukemic acceleration.

Further analyses indicated that *Vav3*^*−*/*−*^ leukemic B-cell progenitors proliferate less than their control counterparts (Fig. 4E and Supplementary Fig. 4A). Bmi1 overexpression results in increased proliferation of both WT and *Vav3*^−/−^ leukemic B-cell progenitors (Fig. 4E and Supplementary Fig. 4A). However, the proliferation of Bmi1-expressing *Vav*3^−/−^ cells is significantly lower (~20% reduction) than the WT controls (Fig. 4E and Supplementary Fig. 4A). This suggest that Bmi1 overexpression cannot overcome the proliferative deficiency exhibited by *Vav3*^−/−^ cells. We also found that the Vav3 deficiency and the Bmi1 overexpression impairs and increases the self-renewal ability of leukemic progenitors, respectively (Fig. 4F). The overexpression of Bmi1 partially rescues the effect of Vav3 deficiency on the frequency of primary clonogenic leukemic progenitors but not the negative effects caused by the Vav3 deficiency in both leukemic progenitor proliferation and self-renewal (Fig. 4F). At the biochemical level, Bmi1 overexpression does not rescue the impaired H2AK119Ub of Vav3-deficient leukemic B-cell progenitors (Fig. 4G). These results indicate that the Bmi1-induced self-renewal, in vivo tumor initiation/propagation and stimulation of PRC1 activity are all Vav3-dependent.

### Bmi1-dependent nuclear Rac activation depends on Vav3

To understand the role of Vav3 in nuclear Rac activation in the context of Bmi1 overexpression, we performed Rac GTPase activation assays in the nuclear fraction of p190-BCR-ABL^+^ B-cell progenitors. Bmi1 overexpression increases while Vav3 deficiency abrogates Rac activation (Fig. 4H). The Bmi1-induced Rac activation in WT leukemic B-cell progenitors is associated with augmented expression and activation of Vav3 (Supplementary Fig. 4B). The genetic ablation of Rac2 GTPase phenocopies the Vav3 deficiency, abrogating the expansion effect of Bmi1 overexpression on p190-BCR-ABL^+^ leukemic B-cell colony formation (Fig. 4I). To further analyze the role of Bmi1 in p190-BCR-ABL^+^ B-cell progenitors in the context of Vav3 and Rac2 deficiencies, we depleted Bmi1 by shRNA-mediated knockdown (Supplementary Fig. 5A). Serial clonogenic CFU-proB assays show that the replating ability of B-cell progenitors transformed by p190-BCR-ABL is completely dependent on endogenous Bmi1 expression (Supplementary Fig. 5B). These data reinforce the role of Vav3 and Rac2 in Bmi1-mediated transformation and self-renewal of leukemic progenitors.

### Vav3 is required to maintain homeostatic PRC1.4 epigenetic programs

**Fig. 5:**
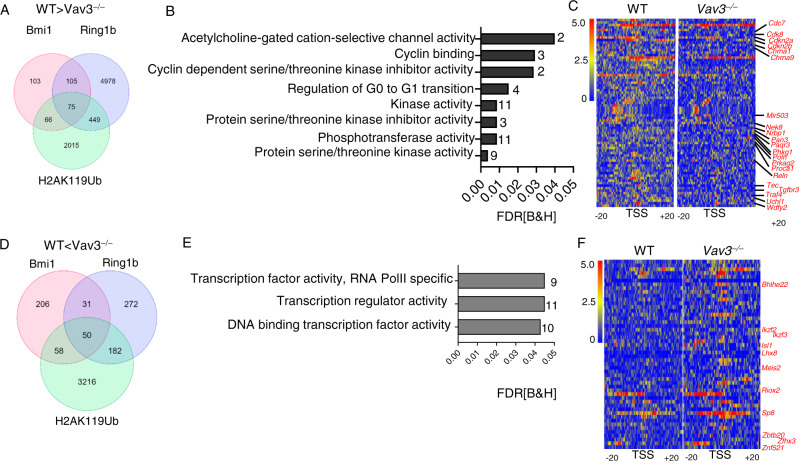
Nuclear Vav3 modulates PRC1 mediated repression of regulators of proliferation and transcriptional factors. **A** Venn diagrams depicting intersection of genes where CUT&RUNseq signal for Bmi1, Ring1b, and H2AK119Ub was decreased in *Vav3*^*−*/*−*^ leukemic B-cell progenitors (Diffbind: Log_2_ Fold Change>1, *p* <  0.05). **B** Gene-ontology analyses of molecular and biological function of the genes with reduced Bmi1, Ring1b, and H2AK119Ub binding. Genes associated with negative regulation of protein kinase activity, negative regulation of protein phosphorylation, and cell cycle G1/S transition show reduced PRC1 components binding. **C** Representative density map of 75 common genes with decreased binding of Bmi1, Ring1b, and H2AK119Ub in *Vav3*^−/−^ leukemic B-cell progenitors. Tracks shown are for Bmi1 binding. Diffbind analyses was performed between two CUT&RUN replicates of WT and *Vav3*^−/−^ leukemic B-cell progenitors, and one representative example density map is presented. **D** Venn diagrams showing intersection of genes where binding of Bmi1, Ring1b, and H2AK119Ub in *Vav3*^*−*/*−*^ leukemic B-cell progenitors was increased (Diffbind: Log_2_ Fold Change>1). **E** Gene-ontology analyses of molecular and biological function of the genes with increased Bmi1, Ring1b, and H2AK119Ub binding. Genes associated with RNA polII transcription regulatory region-specific DNA binding, transcription factor regulator activity show increased PRC1 components binding. **F** Representative density map of 50 common genes with increased binding of Bmi1, Ring1b and H2AK119Ub in *Vav3*^*−*/*−*^ leukemic B-cell progenitors. Tracks shown are for Bmi1 binding. Diffbind analyses was performed between two CUT&RUN replicates of WT and *Vav3*^−/−^ leukemic B-cell progenitors, and one representative example density map is presented.

To evaluate the effects of Vav3 on PRC1.4 activity at the genome-wide level, we performed cleavage under target and release using nuclease followed by next-generation sequencing (CUT&RUN-seq) experiments in WT and *Vav3*^*−*/*−*^ leukemic B-cell progenitors. This technique cleaves DNA sequences that are associated with protein A-conjugated micrococcal nuclease, thus allowing the detection of both protein binding and histone mark enrichments in DNA regulatory regions throughout the whole genome. To this end, we performed CUT&RUN-seq for in WT and *Vav3*^−/−^ leukemic B-cell progenitors using specific antibodies. An anti-Bmi1 antibody, validated to be specific for nuclear Bmi1 as assessed in Bmi1-deleted (CRISPR/Cas9) pre-B leukemic Ba/F3 cells (Supplementary Fig. 6A, B) and antibodies previously validated for chromatin immunoprecipitation followed by next-generation sequencing for Ring1b and H2AK119Ub antibodies^46^ were used. Subsequently, the Bmi1, Ring1b, and H2AK119Ub differential binding in WT and *Vav3*^−/−^ leukemic B-cell progenitors was evaluated as indicated in Methods (Supplementary Figs. 7 and 8). Correlations between replicates of each of the CUT&RUNseq datasets (Supplementary Fig. 7A) confirmed good correlations for anti-Bmi1 binding (*r* ≥ 0.84) with lower correlations for Ring1b and H2AK119Ub marks. We found no overt differences in the distribution of antibody-binding peaks (Supplementary Fig. 7B), although we did detect statistically significant quantitative differences in the mean signal generated by antibody-binding peaks (Supplementary Figs. 7C–E and 8A–C) for Bmi1, Ring1b, and H2AK119Ub. Specifically, 349 loci are significantly depleted of Bmi1 binding in *Vav3*^−/−^ p190-BCR-ABL^+^ B-cell progenitors (Supplementary Fig. 8A and Supplementary Data 4). The differential binding analyses of Ring1b and H2AK119Ub CUT&RUN dataset showed higher number of loci with reduced binding in *Vav3*^*−*/*−*^ leukemic B-cell progenitors (5607 Ring1b bound and 2605 H2AK119Ub bound) (Supplementary Fig. 8B, C and Supplementary Data 5 and 6). To identify the PRC1.4 specific targets, we performed intersection analysis of all three datasets and found 75 common loci (*p* = 1.77 × 10^−49^) including those encoding cell cycle regulators (*Cdkn2a, Cdkn2b, Mir503, Cdc7*, *Cdk8, Nrbp1, Tgfbr3, Phkg1, Nek8, Pan3, Tec, Prkag2, Proca1*) associated with decreased association of Bmi1, Ring1b and H2AK119Ub in *Vav3*^*−*/*−*^ leukemic B-cell progenitors (Fig. 5A–C; Supplementary Fig. 9A, B; and Supplementary data 7). These data further supports the role of Vav3 in the regulation of PRC1.dependent processes such as *Cdkn2a/2b* expression and cell cycle progression described above (Figs. 1H, J and 2B–E, and Supplementary Fig. 10A). They also suggest that the regulation of the foregoing loci at the epigenetic level can represent a plausible mechanism to explain the loss of heterozygosity in leukemic B-cell leukemias which frequently contain heterozygous deletions of only one of *Cdkn2a/b* alleles^16,47^. Conversely, we also found increased Bmi1 presence in 345 loci (Supplementary Figs. 7C and 8A and Supplementary Data 4) as well as a higher number of genes associated with increased Ring1b (535 loci) and H2AK119Ub binding (3506 loci) in cells lacking Vav3 expression (Supplementary Figs. 7D, E and 8B, C and Supplementary Data 5 and 6). Analyses of loci with enhanced Bmi1, Ring1b and H2AK119Ub binding in those cells identified 50 common loci (*p* = 4.5 × 10^−68^) with direct or RNA pol-II binding dependent transcription regulatory activities (Fig. 5D–F, Supplementary Figs. 9C, D and 10B, and Supplementary data 8). We confirmed reduced expression of the tumorigenic transcriptional factors *Isl1*^48^*, Zfhx3*^49^*, Meis2*^50^, and Bhlhe22^51^ *in* Vav3-deficienct cells (Supplementary Fig. 10C), indicating that the increased binding of PRC1.4 complex to these loci is also linked to the repression of these leukemogenic genes in *Vav3*^*−*/*−*^ leukemic B-cell progenitors.

### The Vav3/Phllp2/Akt axis modulates nuclear Bmi1 phosphorylation and PRC1 activity

**Fig. 6:**
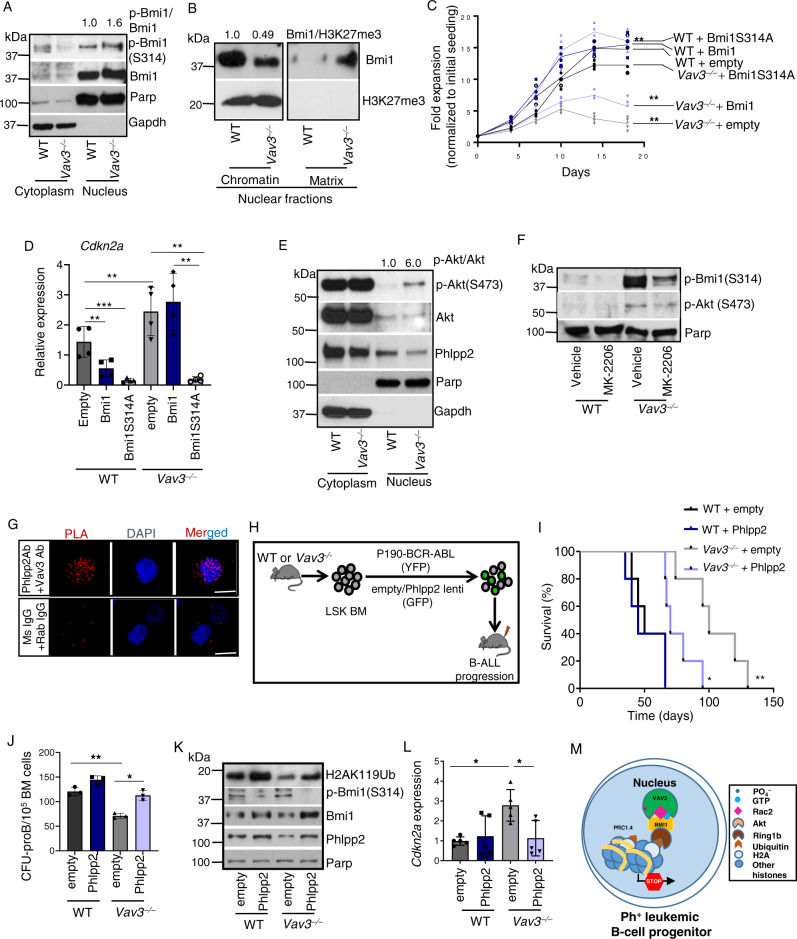
Phlpp2 ectopic expression rescues B-ALL development in *Vav3* deficient B-cell progenitors. **A** Representative immunoblots showing enhanced phosphorylation of Bmi1 in the nuclear fraction of *Vav3*^*−*/*−*^ p190-BCR-ABL^+^ B-cell progenitors. **B** Representative immunoblots showing decreased chromatin bound and increased nuclear matrix Bmi1 in *Vav3*^*−*/*−*^ p190-BCR-ABL^+^ B-cell progenitors. **C** Ex-vivo expansion of mock, Bmi1 and Bmi1 (S314A) transduced WT and *Vav3*^*−*/*−*^ p190-BCR-ABL^+^ B-cell progenitors (*n* = 6 per group). **D** Q-RT-PCR analyses of *Cdkn2a* expression in empty vector/Bmi1/Bmi(S314A) transduced WT and *Vav3*^*−*/*−*^ p190-BCR-ABL^+^ B-cell progenitors (*n* = 4 per group). **E** Representative immunoblots for p-Akt (S-473), Akt and Phlpp2 in the cytoplasmic and nuclear fractions of WT and Vav3-deficient p190-BCR-ABL^+^ B-cell progenitors. Representative membrane blotting is the same as in Fig. 6a. **F** Representative immunoblots for p-Bmi1, pAkt-S473 and Parp in the nuclear fraction of MK2206 treated WT and *Vav3*^*−*/*−*^ leukemic B-cell progenitors. **G** Confocal microscopic images of PLA between Vav3 and Phlpp2 in the nucleus of leukemic B-cell progenitors. **H** Schematic diagram depicting co-transduction of WT and *Vav3*^*−*/*−*^ Lin^−^cKit^+^Sca1^+^ BM cells with p190-BCR-ABL retroviruses and empty or Phlpp2 lentiviruses followed by transplantation into C57bl/10 mice. **I** Kaplan-Meier survival analyses of primary recipient mice transplanted with WT or *Vav3*^−/−^ Lin^−^cKit^+^Sca1^+^ co-transduced with p190-BCR-ABL retroviruses and empty or Phlpp2 lentiviruses (*n* = 5 mice per group). Vav3 deficiency significantly prolongs the survival. Phlpp2 overexpression in *Vav3*^*−*/*−*^ progenitors restores leukemogenesis. **J** CFU-proB content of empty vector or Phlpp2 transduced WT and *Vav3*^*−*/*−*^ leukemic B-cell progenitors (*n* = 3 per group). **K** Representative immunoblots for p-Bmi1, Bmi1, H2AK119Ub, Phlpp2, and Parp in WT and *Vav3*^*−*/*−*^ leukemic B-cell progenitors transduced with empty or Phlpp2 lentiviruses. **L** Q-RT-PCR analyses of *Cdkn2a* expression in empty or Phlpp2 transduced WT and *Vav3*^*−*/*−*^ leukemic B cell progenitors (*n* = 5 per group). **M** Schema representing the nuclear protein complex controlling leukemogenic PRC1.4 activity. Scale bar, 10 μm. Data are presented as mean ± SD of two or three independent experiments. Statistical significance was determined using the unpaired Student-*t* or Anova tests when more than two groups were compared. Differences in survival were examined using the log-rank P test. *p <  0.05; **p <  0.01; ***p <  0.001.

It has been shown that the phosphorylation of Bmi1 on S^314^ by activated Akt leads to the disassembly of Bmi1 from PRC1 complexes and the ensuing de-repression of *Cdkn2a*^52^. To understand how the Vav3 deficiency ablates the oncogenic effect of Bmi1, de-represses the genes associated with proliferation and differentiation, and results in the rewiring of Bmi1 binding to genes involved in self-renewal, we next decided to compare the expression and phosphorylation levels of Bmi1 in p190-BCR-ABL^+^ WT and *Vav3*^−/−^ B-cell progenitors. Using these analyses, we found that the loss of Vav3 results in a 59 ± 4.9% (*p* < 0.01) increase in phospho-S^314^ Bmi1 protein levels in the nuclear fraction of p190-BCR-ABL^+^ *Vav3*^−/−^ B-cell progenitors (Fig. 6A). Although total Bmi1 expression remains unchanged, Vav3 deficiency was associated with shuttling of Bmi1 from being bound to chromatin to the nuclear matrix (Fig. 6B). This increased phosphorylation level correlated with enhanced expression of *Cdkn2a, Cdkn2b*, *and Pax5* transcripts in *Rac2*^−/−^ leukemic B-cell progenitors (Supplementary Fig. 11A, B). Together, these data indicate that Vav3–Rac2 activity is required to ensure nuclear expression and chromatin binding of Bmi1.

To further examine if the deficiency of VAV3 leads to increased BM1-1 phosphorylation and growth arrest of human B-cell precursor leukemias, we silenced *VAV3* in four independent Ph^+^ human B-precursor leukemia cell lines (BV-173, NALM-1, TOM-1, and SUP-B15)^53–56^. These cell lines contain additional secondary mutations in addition to those targeting the BCR and ABL loci. Similar to primary cells, we found that VAV3 expression is predominantly nuclear in these B-ALL cell lines (Supplementary Fig. 12A). More importantly, we found that upon downregulation of VAV3, the residual expression of VAV3 inversely correlates with BMI1 phosphorylation (Supplementary Fig. 12B). The silencing of VAV3 also leads to reductions in the frequency of CFU-blast formation and the ability to expand in liquid culture (Supplementary Fig. 12C, D). To verify that the Vav3-Rac2 axis is involved in the down-modulation of the dephosphorylated status of Bmi1, we transduced WT and *Vav3*^−/−^ leukemic B-cell progenitors with either wild-type or a phosphorylation-deficient version (S314A) of Bmi1 using a lentiviral delivery method (Supplementary Fig. 13A). As anticipated, the expression of Bmi1(S314A) rescues the leukemic cell growth (Fig. 6C), H2AK119 ubiquitination (Supplementary Fig. 13A), and the repression of *Cdkn2a* of *Vav3*^−/−^ p190 BCR-ABL^+^ B-cell progenitors (Fig. 6D).

To further understand this process, we analyzed the levels of activated Akt (phospho-S^473^) in the cytoplasmic and nuclear fractions of both WT and *Vav3*^−/−^ leukemic B-cell progenitors. We observed that Akt preferentially localizes in the cytoplasmic fraction regardless of the genotypes of the cells analyzed (Fig. 6E). However, we found 6.0 ± 1.1 higher levels of activated Akt in the nuclear fraction of *Vav3*^−/−^ leukemic B-cell progenitors when compared to the wild-type controls (*p* <  0.001; Fig. 6E). The allosteric Akt specific inhibitor MK2206 reduces Bmi1 phosphorylation found in Vav3-deficient cells (Fig. 6F), indicating that the enhanced expression of phospho-Bmi1 found in these cells depends on nuclear Akt activation. Akt activation is downregulated by the concurrent action of the protein phosphatase 2A (Pp2a) and the pleckstrin homology domain leucine-rich repeat protein phosphatase 2 (Phlpp2) that target the phosphorylated T^308^ and S^473^ residues of Akt, respectively^57,58^. We observed reduced levels (40 ± 2%, *p* <  0.01) of Phlpp2 in the nuclear fraction of *Vav3*^−/−^ leukemic B-cell progenitors (Fig. 6E). This change is not observed at the transcript level (Supplementary Fig. 13B), indicating that this phosphatase is regulated at the posttranscriptional level. The expression of Pp2a is also reduced in the absence of Vav3 (41 ± 15%, *p* < 0.05, *n* = 2). However, in this case, we could not observe any variation in the levels of phospho-Akt(T308) in *Vav3*^−/−^ leukemic progenitor nuclear fraction (Supplementary Fig. 13C). This suggest that Pp2a is not likely involved in this regulatory step. Further strengthening the signaling connection between Vav3 and Phlpp2, we found that these two proteins are in close nuclear proximity (Fig. 6G). Such a proximity is not found between Bmi1 and Phlpp2, indicating that it is unlikely that Phlpp2 itself regulates Bmi1 phosphorylation (Supplementary Fig. 13D). These data support the concept that Vav3 associates with Phlpp2 to maintain low levels of Akt and Bmi1 phosphorylation in the nucleus of B-ALL progenitors.

### Ectopic expression of Phlpp2 restores nuclear Bmi1 expression and leukemogenesis of Vav3-deficient leukemic B-cell progenitors

To understand the role of Phlpp2 in Vav3-dependent B cell leukemogenesis, we isolated Lineage^−^/c-Kit^+^/Sca-1^+^ (LSK) bone marrow progenitors from WT or *Vav3*^−/−^ mice that, after transductions with p190-BCR-ABL-encoding retroviruses plus either control or Phlpp2-expressing lentiviruses, were transplanted into C57/Bl10 congenic recipients (Fig. 6H). Mice transplanted with BCR-ABL^+^ LSK cells transduced with Phlpp2-expressing and control lentiviral particles develop B-ALL with a median survival of 50 and 45 days, respectively (Fig. 6I). As expected, mice transplanted with *Vav3*^−/−^ LSK co-transduced with p190-BCR-ABL and empty vector survive significantly longer (median survival 100 days) than the previous ones. However, the ectopic expression of Phllp2 in those cells abrogates the effect of the Vav3 deficiency, leading to a significant shortening of the median time of death down to 70 days (Fig. 6I). The overexpression of Phlpp2 also restores the clonogenicity of *Vav3*^–/–^ leukemic B-cell progenitors (Fig. 6J) as well as normal levels of Bmi1 phosphorylation, H2AK119 ubiquitination, and *Cdkn2a* and *Pax5* expression (Fig. 6K, L and Supplementary Fig. 13E). These results demonstrate that the nuclear Vav3–Phllp2–Akt–Bmi1 axis plays essential roles in the progression of p190-BCR-ABL^+^ B-ALL (Fig. 6M).

## Discussion

In the present study, we demonstrate that Vav3 becomes unexpectedly upregulated and activated in the nucleus of both human and murine lymphoblastic leukemia progenitors upon expression of BCR-ABL. Furthermore, we have shown that the exchange activity of Vav3 found in the nucleus is important for the proliferation and progression of p190-BCR-ABL^+^ leukemia using a PRC1- and to a lesser extent PRC2-dependent mechanism. Leukemic B-cell progenitors lacking Vav3 have impaired PRC1.4 activity associated with increased phosphorylation and inactivation of Bmi1. We have previously reported the anti-apoptotic activity of VAV3^30^ and its targeting by a first-in-class small molecule inhibitor which significantly impairs human and murine B-cell progenitor lymphoblastic leukemogenesis in vitro and in vivo^31^. Together, these data consolidate our understanding of VAV3-driven oncogenesis and their translational importance for B-ALL therapy.

The reduced proliferation of Vav3-deficient lymphoid leukemia B-cell progenitors is associated with increased expression of at least *Cdkn2a* and *Cdkn2b*, genes known to be regulated by PRC. The PRC system (PRC1 and PRC2) regulates developmental gene expression, control stem cells self-renewal and lineage commitment, and are frequently deregulated in cancer^59^. Both PRC complexes work in tandem to regulate histone modification and repression of target genes. The classical mode of gene repression commences with H3K27 tri-methylation by PRC2 followed by PRC1 mediated H2AK119 mono-ubiquitination resulting in repression of gene expression. PRC1 complex is formed by the association of RING1A or RING1B with one of six PCGF proteins (PCGF1-6) forming PRC1 through PRC6 complexes with distinct biological functions^60^. BMI1/PCGF4, the critical component of the PRC1.4 controls the proliferative capacity and self-renewal ability of normal and leukemic stem cells^20,21^. BMI1 repressive activity requires PRC2/EZH2-dependent H3K27me3^44,61^ and activation of E3-ubiquitin ligase activity of RING1 proteins to mono-ubiquitinate H2A (K119)^45^. We also identified a set of recognized oncogenic transcriptional factors whose expression is further repressed by PRC1.4 in Vav3-deficient leukemic B-cell progenitors. Together, this set of data strongly suggest that Vav3-dependent transcriptional repressive program is not universal, with a number of loci in which Vav3-dependent PRC1.4 repressive activity is probably replaced by alternative systems that activate PRC1.4 repressive activity in absence of Vav3.

BMI1 is upregulated in patients with advanced stages of BCR-ABL-induced chronic myelogenous leukemia (CML)^8^ and through its repressive activity on the *Cdkn2a* locus^18^ and other less well-characterized *Cdkn2a* independent activities results in leukemic transformation^19^. Co-expression of BMI1 and BCR-ABL in human cord blood CD34^+^ cells induces leukemia that can be propagated serially in immunodeficient mice with a bias towards lymphoid blast crisis^9^. Our group has demonstrated that BMI1 transforms and reprograms CML B-lymphoid progenitors into self-renewing, leukemia initiating cells^10^. Bmi1 deletion accelerates B-cell differentiation through upregulation of lymphoid specification genes^62^ and the transcriptional repressor activity of Bmi1 is required for normal and leukemic stem cell self-renewal^20,21^.

Bmi1 phosphorylation on residue S314 by Akt^52^ leads to its inactivation and its dissociation from- the PRC1 complex resulting in de-repression of *Cdkn2a* locus^52^. This effect is residue specific because the phosphorylation of Bmi1 on other Akt-dependent sites S^251^, S^253^, and S^255^ results in Bmi1 activation^63^. We found increased Bmi1 phosphorylation on S314 in the absence of expression of Vav3 or Rac2, indicating that Vav3/Rac2 prevent Bmi1 phosphorylation. Increased phosphorylation of Bmi1 is associated with increased Akt activation in the nucleus and reduced level of phospho-Akt(S473) specific protein phosphatase Phlpp2^64^. Phlpp2 was found not to be in direct proximity to Bmi1 suggesting that Phlpp2 is unlikely to be acting directly on Bmi1 phosphorylation. Overexpression of Phlpp2 or the mutant Bmi1 (S314A), but not wild-type Bmi1, results in the restoration of leukemogenesis and transcriptional repression. The mechanisms by which Vav3 controls the nuclear expression of the tumor suppressor Phlpp2 remain unclear but Phlpp2 stabilization by p27(kip1), a cyclin-dependent kinase inhibitor upregulated by BCR-ABL in leukemic progenitors^65^, has been highlighted^66^ and it is possible that Vav3 participates in the stabilization of Phlpp2.

Nuclear Rac plays a critical role in chromatin modifications and gene expression^67^ and downstream actin polymerization plays a major role in gene relocalization between heterochromatin and euchromatin that controls differentiation of embryonic stem cells^68^. Rac and its downstream target Wave are required for transcriptional reprogramming^69^ and polymerized actin has been shown to control polycomb-mediated gene silencing^70^. In leukemic progenitors, Rac2 suffices to phenocopy the effect of Vav3 deficiency while Rac1 is dispensable for BCR-ABL-induced proliferation^22,23,30^. Along with our published data^30^, this report supports a selective role for nuclear Vav3 and Rac2 in the regulation of PRC1.4 dependent transcription repression and reprogramming in B-cell leukemogenesis and suggests they control higher level nuclear structures of transcriptional repression with selectivity to act on specific loci. Anti-Bmi1 binding allowed us to establish sufficient signal/noise ratio to distinguish the most differentially bound loci in Vav3-deficient leukemic progenitors and identify 75 loci with reduced PRC1.4 binding/activity relevant to proliferation and differentiation of B-cell progenitors while other 50 loci, relevant in self-renewal, have increased PRC1.4 binding/activity. The mechanisms of this differential binding are unclear but it may reside on the selectivity of high-order chromatin structures for preferential binding and the use of alternative, non-canonical PRC1.4 complexes (reviewed in ref. ^71^).

In summary, the results of the present study show that Vav3 predominantly resides in the nucleus of leukemic B-cell progenitors where it plays a pivotal role in Ph^+^ leukemogenesis by modulating PRC1.4 activity and is dependent on nuclear Phlpp2-regulated Akt activity. This description of a nuclear GEF activity associated with an oncogenic polycomb repression program, opens a whole area of research to better understand the role of nuclear Rho GTPases in leukemogenesis.

## Methods

### Animals

The generation of Vav3-deficient (*Vav3*^*−*/*−*^) mice^72^ and Rac2-deficient (*Rac2*^−/−^) mice^73^ have been described previously. All mutant mice were backcrossed >10 generations into C57Bl/10 or C57Bl/6 mice, respectively. To avoid possible interference with androgen signaling, 6- to 8-week-old female wild-type (WT) C57Bl/10 and C57Bl/6 mice were obtained commercially (Jackson Laboratory, Ban Harbor, ME and Harlan Laboratories, Indianapolis, IN, respectively) and used as donors and/or recipients for transduction/transplantation models. All mouse strains were maintained at an Association for Assessment and Accreditation of Laboratory Animal Care accredited, specific-pathogen–free animal facility at Cincinnati Children’s Research Foundation, Cincinnati, under an Institutional Animal Care and Use Committee approved protocol. The transgenic mice used in the study were between 6 and 12 week of age at the time of experimentation.

### Human specimens

Four B-ALL patient samples were obtained from The Pediatric Leukemia Avatar Program within the Cancer & Blood Diseases Institute (CBDI) at Cincinnati Children’s Hospital Medical center, under IRB approval and informed consent. All leukemic specimens contained more than 80% blasts and corresponded to specimens obtained at diagnosis or relapse. Genotyping characterization of these four leukemias had the following monoallelic mutations:

UID 2016-11: BCR/ABL, BCR/EXOSC2, CDKN2A loss, CDKN2B intron 1 truncation;

UID 2017-129: BCR/ABL T315I, CD36 splice site 609+1G>A, SETD2 E282fs*19, SF3B1 T663I (sub), TLL2 G891fs*3, TP53 R248Q.

UID 2018-210: BCR/ABL F359V, T315I, CDKN2A/B loss, IKZF1 loss, MLL2 S2173*, PAX5 Y179fs*64.

UID 2018-132: JAK1 L653 (subclonal) and R724H (subclonal), JAK2 R867Q, IGH/CRLF2, CDKN2A loss exon 1, CDKN2B loss exon 2, FOXP1 R544*, ZRSR2 R448_R449insSRSR.

The B-ALL B-cell progenitors (hCD45^+/low^, hCD34^+^, hCD19^+^) cells from these leukemic specimens were sorted and used for confocal immunofluorescence microscopy, western blot and quantitative real time PCR analyses.

Human leukemic cell lines Bv-173, NALM-1, TOM-1, SUP-B15 were obtained from the German Collection of Microorganisms and Cell Cultures (DSMZ; Bv-173, NALM-1 and TOM-1) and American Tissue Culture Collection (ATCC; SUP-B15).

### Viral transduction, progenitor transplantation and development of B-cell acute lymphoblastic leukemia

The method of retroviral vectors generation and transduction have been described previously^22^. In brief, Ba/F3 or mouse LDBM (obtained by Histopaque density gradient, density <1083 g/cm^3^) were transduced with bicistronic retroviral vectors^74^ encoding p190-BCR-ABL (MSCV-p190-BCR-ABL-IRES-EYFP) or empty vector in the presence of 10 ng/mL of recombinant human IL-7 (PeproTech), 50 ng/mL of recombinant mouse SCF (PeproTech) in six-well non-tissue culture plate precoated with recombinant fragment of fibronectin, CH296 (Takara Bio) for 8 h at 37 °C. Mouse LDBM cells were pre-stimulated O/N in presence of 10 ng/mL of recombinant human IL-7, 50 ng/mL of recombinant mouse SCF before adding retroviruses. For the Vav3 rescue experiments, WT or *Vav3* mouse LDBM cells were transduced with MSCV-p190-BCR-ABL-IRES-EYFP vector followed by second round of transduction with lentiviral particles encoding either WT Vav3 (full-length; FL), Vav3 CA (constitutively activated, with the calpain homology CH domain deleted), Vav3 NA (N369A mutant which loses the GEF activity) cloned in a *pCDH-EF1*-MCS-*IRES*-GFP vector (System Biosciences). The empty lentiviral vector without any cDNA was used as control. For Bmi1 ectopic expression in combination with p190-BCR-ABL, WT or *Vav3*^−/−^ LDBM cells were co-transduced with MSCV-p190-BCR-ABL-IRES-EYFP retroviruses and Bmi1-IRES-EGFP or IRES-EGFP (empty) lentiviral vectors (kindly provided by Dr. Sally Temple, Neural Stem Cell Institute, Rensselaer, NY)^75^. For Bmi1(S314A) ectopic expression in combination with p190-BCR-ABL, WT or *Vav3*^*−*/*−*^ LDBM cells were co-transduced with MSCV-p190-BCR-ABL-IRES-EYFP retroviruses and Bmi1(S314A)-IRES-EGFP or IRES-EGFP (empty) lentiviral vectors. The EYFP^+^EGFP^+^ B-cell progenitors were sorted and cultured in vitro in presence of mSCF (50 ng/mL) and mIL-7 (10 ng/mL). For Phlpp2 rescue experiments, Lin^−^cKit^+^Sca1^+^ cells derived from WT or *Vav3*^−/−^ mice were transduced with MSCV-p190-BCR-ABL-IRES-EYFP retroviruses for 8 h followed by a second round of transduction with lentiviral vector EF1a-Phlpp2-IRES2-EGFP (pReceiver-Lv165-Phlpp2) or empty vector (EF1a-IRES2-EGFP, pReceiver-Lv165) purchased from Genecopoeia. For Bmi1 down regulation, mouse LDBM cells derived from WT, *Vav3*^−/−^ and *Rac2*^−/−^ mice were co-transduced with MSCV-p190-BCR-ABL-IRES-EYFP and a validated Bmi1 shRNA against Bmi1 cDNA (NM_0075521-146s1c1; CCGGCCAGCAAGTATTGTCCTATTTCTCGAGAAATAGGACAATACTTGCTGGTTTTT) expressed in a pLKO.1-puro-CMVtGFP lentiviral vector (Sigma-Aldrich) at a multiplicity of infection of 20.

LDBM cells from WT or *Vav3*^−/−^ mice were isolated and transduced with either a retroviral bicistronic vector encoding p190-BCR-ABL with enhanced yellow fluorescent protein (EYFP) and lentiviral vector expressing Bmi1 or Vav3 mutants or Phllp2 with enhanced green fluorescent (EGFP) as a reporter or an empty vector solely expressing the reporter protein (empty). Transduced LDBM cells (10^6^ per mouse) were intravenously transplanted into lethally (7 + 4.75 Gy) irradiated C57Bl/10 female mice. The multiplicity of infection of p190-BCR-ABL retroviral vector was kept low (at a level of 1) and transduction efficiencies of ~10% were found in all groups, to guarantee integration of one single copy. Mice were followed for the development of B-cell acute lymphoblastic leukemia. The sick and moribund mice were killed, and bone marrow and spleen were collected for leukemia analyses. For secondary transplantation, lethally irradiated recipient mice were intravenously transplanted with 4 × 10^5^ sorted EGFP^+^ B220^+^cKit^+^ BM cells along with 1 × 10^6^ normal, congenic C57Bl/10 BM cells. For limiting dilution transplantation experiments, p190-BCR-ABL^+^ B-cell progenitors derived from WT or *Vav3*^−/−^ primary leukemic mice were transplanted at different doses (For each mouse: 10^5^, 3 × 10^4^ and 10^4^) along with 1 × 10^6^ normal C57Bl/10 female BM cells. Congenic C57/Bl10 BM cells intravenously into lethally irradiated secondary C57/Bl10 recipients. Mice were followed for the development of B-cell acute lymphoblastic leukemia. The sick and moribund mice were killed, and bone marrow and spleen were collected for leukemia analyses.

For xenotransplantation of Ph^+^ and Ph^+^ (T315I) mutant leukemia analyses, patient derived B-cell progenitors (CD34^+^/CD19^+^) were transduced with our previously validated *VAV3* shRNA lentiviral vectors^30^ followed by transplantation into NOD-*scid* IL2Rgamma^null^ (NSG) humanized mice model (Fig. 1K). The mice were killed at 5 weeks post transplantation and EGFP^+^ human chimera in the BM were analyzed by flow cytometry.

### Flow cytometry, Immunophenotypic analysis, and cell sorting

For the analyses of p190-BCR-ABL^+^ (EYFP^+^) leukemic B-cell progenitors, the PB, BM, and splenocytes of primary and secondary recipient leukemic mice were stained using APC-Cy7–anti–mouse CD45R (clone RA3-6B2, Catalog 552094, BD-Pharmingen), PerCP-Cy5.5–anti–mouse IgM (clone II/41, Catalog 550881, BD-Pharmingen), PE–anti–mouse CD43 (clone S7, Catalog 553271, BD-Pharmingen), and PE-Cy7- anti-mouse CD19 (clone 1D3, Catalog 552854, BD-Pharmingen) antibodies. For the immunophenotypic characterization of p190-BCR-ABL+ leukemic B-cells progenitors in mock/Bmi1 transduced and transplanted mice, the PB, BM and splenocytes were stained for APC-Cy7–anti–mouse CD45R (clone RA3-6B2), PerCP-Cy5.5–anti–mouse IgM (clone II/41), PE–anti–mouse CD43 (clone S7), PE-Cy7- anti-mouse CD19 antibodies, and EGFP^+^ EYFP^+^ B-cell progenitors were analyzed. FACS-Canto flow cytometer and the FACSArial cell sorter (both BD Biosciences) were used for analyses and sorting, respectively. For the BM chimera analysis of NSG mice transplanted with Ntg or *VAV3* shRNAs (EGFP^+^) transduced human B-ALL, BM cells were stained for V450-hCD45 (clone HI30, Catalog 560367, BD Horizon), PE-hCD34 (clone 581, Catalog 555822, Biolegend), and APC-Cy7-hCD19 (clone SJ25C1, Catalog 557791, BD-Pharmingen). All antibodies were used at a dilution of 1:100 for 1 × 10^6^ cells.

### CFU-proB assay

B-cell lineage colony-forming units (CFU-proB) of efficiency WT, *Vav3*^*−*/*−*^and *Rac2*^−/−^ p190-BCR-ABL^+^ B-cell progenitor transduced with empty or Bmi1 or Phlpp2 lentiviral vector were quantified after 9 days culture of leukemic BM cells or sorted p190-BCR-ABL expressing B-cell progenitors in M3134 methylcellulose (Stem Cell Technologies, Vancouver, BC) containing 20 ng/mL of recombinant mouse IL-7 and 100 ng/mL of recombinant mouse SCF (PeproTech, Rocky Hill, NJ) and supplemented with 30% FBS (Hyclone GE Healthcare, Marlborough, MA), 2 mM l-glutamine (Invitrogen, Waltham, MA), 1% antibiotics (penicillin-streptomycin; Invitrogen), 100 μM β-mercaptoethanol (Thermo Fisher Scientific, Waltham, MA), and 1% BSA (Sigma-Aldrich, St. Louis, MO). CFU-proB containing clusters of more than 40 cells were counted on day 9 of culture.

### Proliferation and survival assays

Proliferation and Survival of B-cell progenitors were determined by in vivo BrdU uptake and annexin V binding assay, respectively. For in vivo proliferation analysis, mice were intraperitoneally injected 500 μg of BrdU. Forty-five minutes later, BM cells were harvested and stained for surface markers for the identification of B-cell progenitors (B220-APC-Cy7; CD19-PE-Cy7; CD43-PerCP Cy5.5). The cells were fixed, permeabilized, and stained with APC-conjugated anti- BrdU antibody (α-BrdU-APC) according to the manufacturer’s protocol (BD Biosciences, San Jose, CA) and then FACS analyzed using a FACS Canto II analyzer (BD Biosciences). For in-vitro expansion of p190-BCR-ABL^+^ WT, *Vav3*^*−*/*−*^ leukemic cells transduced with mock or Bmi1 or Bmi1(S314A) lentiviral vectors were cultured in 24-well plate, and cell growth was counted every 3rd or 4th day following initial seeding, and plotted. To evaluate the survival, freshly isolated BM cells from leukemic mice were stained for cell surface markers for the identification of B-cell progenitors (B220-APC-Cy7; CD19-PE-Cy7; CD43-PerCP Cy5.5) and APC-conjugated annexin V Ab for 30 min at RT. Cells were washed to remove unbound antibodies, and FACS analyzed using a FACS Canto II analyzer (BD Biosciences).

### Western blot analyses and co-immunoprecipitation

B-cell progenitors (FSC^hi^ B220^lo^ IgM^−^ CD43^+^ CD19^+^) were sorted from BM cells of primary leukemic mice belonging to WT, *Vav3*^−/−^, *Rac2*^−/−^ transduced with empty or Bmi1 or Phlpp2 lentiviral vectors. B-cell progenitors (hCD45^+/lo^hCD34^+^hCD19^+^) from human B-ALL patient samples were FACS sorted. The EGFP+ cells were sorted from Ntg shRNA or VAV3 shRNAs lentiviral transduced B-ALL cell line Bv-173 and NALM1. Freshly sorted murine progenitors, human B-cell progenitors, and VAV3 (or non-targeted, NTG) shRNA transduced B-ALL cell lines were processed for cytoplasmic and nuclear fractionation using NE-PER Nuclear and Cytoplasmic extraction reagent as per manufacturer’s instruction (ThermoFisher Scientific). For nuclear fractionation, isolated nuclei were treated with chromatin isolation buffer (30 mM Tris-HCl, pH7.9, 200 mM NaCl, 0.5 mM EDTA, 0.5% Triton-X 100 containing Protease inhibitor cocktail), lysed by pipette up and down, incubated on ice for 10 min, and then centrifuged for 5 min (8000 × *g*, 4 °C). The pellet contained the chromatin fraction and the supernatant contained the nuclear matrix which was depleted of chromatin. The cytoplasmic and nuclear fractions were dissolved in Laemmli buffer, boiled for denaturation, and electrophoresed through 4–15% SDS-PAGE gradient gel followed by transfer to PVDF membrane. The membranes were blocked and treated with primary antibodies against Vav3/VAV3 (rabbit polyclonal, from Dr. Bustelo’s laboratory, dilution 1:1000 v/v), phosphoVav3-Y173 (Abcam; Catalog ab109544, dilution 1:1000 v/v), Bmi1 (EMD Millipore; Catalog 05-637, 1:1000: Cell signaling technologies, Catalog 5856, dilution 1:1000 v/v), phosphoBmi1-S314 (rabbit polyclonal, from Dr. Nimer’s laboratory, dilution 1:1000 v/v), Ring1B (Cell signaling technologies; Catalog 5694, dilution 1:1000 v/v), Ezh2(Cell signaling technologies; Catalog 4905, dilution 1:1000 v/v), Phllp2 (Abcam; Catalog ab71973, dilution 1:2000 v/v), PP2A-subunit C (Cell signaling technologies; Catalog 2038, dilution 1:1000 v/v), phosphoAkt-S473 (Cell signaling technologies; Catalog 9018, dilution 1:1000 v/v), phosphoAkt-T308 (Cell signaling technologies; Catalog 13038, dilution 1:1000 v/v), Akt (Cell signaling technologies; Catalog 2967, dilution 1:1000 v/v), Gapdh (Cell signaling technologies; Catalog 5174, dilution 1:1000 v/v), Parp (Cell signaling technologies; Catalog 9532, dilution 1:1000 v/v), β-actin (Sigma-Aldrich; catalog A5441, dilution 1:5000 v/v), followed by washing and subsequent treatment with secondary antibodies tagged with HRP (anti–mouse IgG, catalog 7076, Cell Signaling Technologies; anti–rabbit IgG, catalog 7074, Cell Signaling Technologies, dilutions 1:10,000 v/v). The blots were developed using a chemiluminescence coupled reaction. The band intensities on the X-ray films were quantitated by using ImageJ software (ImageJ V1.5.3), and normalized against Actin, Gapdh or Parp band intensity of the corresponding sample.

For co-immunoprecipitation, the cytoplasmic and nuclear fractions were incubated with α-Vav3 Ab or α-Bmi1 Ab O/N with constant shaking at 4 °C. The lysates were added with 10 μL protein A/G Dynabeads (ThermoFisher; Catalog 10015D) and incubated for 2 h with constant shaking. The samples were washed 3 times with 1X RIPA buffer (Cell Signaling Technologies, Catalog 9806) containing protease inhibitor cocktail (Roche, Catalog 04693159001) and phosphatase inhibitors (Roche, Catalog 046906837001) using a magnetic stand. The immunoprecipitated samples were dissolved in Laemmli’s buffer, boiled for denaturation, and then samples were electrophoresed through 4–15% SDS-PAGE gradient gel followed by transfer to PVDF membrane. The PVDF membranes were subjected to western blot analyses as described above.

### Confocal immunofluorescence microscopy and proximity ligation assay

Sorted murine B-cell progenitors derived from WT or *Vav3*^−/−^ leukemic mice and human B-cell progenitors from B-ALL patient samples were seeded onto fibronectin (RetroNectin catalog T100B, Takara Bio INC.) coated glass chamber slide in culture medium containing mouse or human stem cell factor (50ng/mL) and IL7 (10 ng/mL). Cells were allowed to adhere to the fibronectin coated glass chamber slide for 1 h, and then fixed using 4% paraformaldehyde for 30 min at 4 °C, permeabilized with 0.1% Triton X-100 (catalog T9284, Sigma-Aldrich) for 10 min followed by blocking with 5% normal goat serum in PBS for 30 min. The slides were stained with primary antibodies; α-Vav3 rabbit polyclonal for mouse cells (generated by Dr. Bustello’s Laboratory, dilution 1:250 v/v) or α-VAV3 (rabbit polyclonal, EMD Millipore; catalog 07-464, dilution 1:200 v/v) for human cells at 4 °C overnight. The control or Bmi1-deleted (CRISPR/Cas9) pre-B B-ALL Ba/F3 cells were stained for Bmi1 using α−Bmi1 rabbit monoclonal antibody (Cell signaling technologies, Catalog 5856, dilution 1:50 v/v) at 4 °C overnight. The cells were washed and then treated with secondary antibodies (from Life technologies) donkey anti-rabbit Alexa Fluor 488 (catalog A11034) or donkey anti-rabbit Alexa Fluor 568 (catalog A10042) at 1:500 v/v concentration for 1 h at room temperature. Cells were washed and mounted using Gold Antifade mounting media (catalog P36935, Life technologies) containing DAPI. The stained cells were analyzed by a LSM 710 confocal microscope system (Carl Zeiss) equipped with an inverted microscope (Observer Z1, Zeiss) using a Plan Apochromat ×63 1.4 NA oil immersion lens. The images were processed using Adobe Photoshop CC2019. For the evaluation of VAV3 cellular distributions in nucleus and cytoplasm, the confocal images were processed using Imarisx64 9.6.0 software by creating a 3D surface corresponding to DAPI staining region followed by removal of VAV3 fluorescence signals outside or inside of the DAPI stained nucleus, and presented as ratio of nuclear exclusive and cytoplasmic exclusive VAV3 expressions.

Proximity ligation assay (PLA) was performed to detect interaction of protein residing in close proximity or in a multi protein complex, as per manufacturer’s instruction (Sigma-Aldrich; catalog DUO92002, DUO92004). Briefly, cells were adhered to fibronectin-coated glass chamber slides were fixed using 4% paraformaldehyde, permeabilized with 0.1% Triton X-100 for 10 min, blocked with DuoLink Blocking solution supplied with DuoLink PLA assay kit (Sigma-Aldrich; catalog DUO92002, DUO92004) followed by primary antibody treatment [rabbit α-Vav3 Ab, dilution 1:250 v/v and mouse α-Bmi1 Ab (EMD Millipore, Catalog.05637, dilution 1:200 v/v) or goat α-Phlpp2 Ab (Abcam, Cataog. Ab62830, dilution 1:200 v/v) and rabbit α-Vav3 Ab or rabbit α−Vav3 Ab and mouse α−cAbl Ab (Invitrogen, Catalog. 41–2900, dilution 1:200 v/v) or rabbit α–Phlpp2 Ab (Abcam, Catalog. ab71973, dilution 1:200 v/v) or rabbit α−PP2A Ab (Cell signaling technologies, Catalog 2038, dilution 1:100 v/v) and mouse α−Bmi1 Ab (EMD Millipore, Catalog.05637, dilution 1:200 v/v) in DouLink antibody diluent solution supplied with the kit. Human B-ALL B-cell progenitors were stained with α-VAV3 Ab (EMD Millipore, Catalog. 07–464, dilution 1:200 v/v). Cells were washed, treated with secondary antibody coupled with nucleotide probes (DuoLink InSitu PLA probe α-Rabbit PLUS and DuoLink InSitu PLA probe α-mouse MINUS or DuoLink InSitu PLA probe α-rabbit PLUS and DuoLink InSitu PLA probe α-Goat MINUS) for 1 h at 37 °C in a humidified chamber followed by ligation (30 min at 37 °C), washing and amplification using amplification mix containing detection reagent (100 min at 37 °C), as per manufacturer’s instructions. Slides were mounted in mounting media containing DAPI (supplied with DuoLink InSitu PLA mounting media with DAPI). The stained cells were analyzed by a LSM 710 confocal microscope system (Carl Zeiss) equipped with an inverted microscope (Observer Z1, Zeiss) using a Plan Apochromat × 63 1.4 NA oil immersion lens. The images were processed using Adobe Photoshop CC2019 v20.

### Rac activation assays

WT and *Vav3*^−/−^ p190-BCR-ABL^+^ B-cell progenitors transduced with empty or Bmi1 lentiviral vector were processed for the evaluation of activated Rac and Ras GTPases in the nuclear fraction. Freshly isolated cells were subjected to cytoplasmic and nuclear fractionation. The nuclear fractions were diluted (1:1) with 2x Magnesium Lysis/Wash Buffer (Upstate Biotechnology, Lake Placid, NY) supplemented with 25 mM NaF and 1 mM Na_3_VO_4_. GTP-bound Rac from the lysates was immunoprecipitated using PBD (Pak1 binding domain) agarose beads (Upstate Biotechnology) agarose beads, according to the manufacturer’s instructions. The agarose bead pellets containing GTP bound Rac GTPase were separated by sodium dodecyl sulfate (SDS)-polyacrylamide gel electrophoresis (PAGE) on a 12% gel (Bio-Rad, Hercules, CA), transferred to PVDF membrane followed by western blot analyses using antibodies against Rac GTPase (BD transduction laboratories, Catalog 610650; dilution 1:1000 v/v). Membranes were incubated with primary antibodies overnight at 4 °C, followed by treatment with secondary antibody conjugated to horseradish peroxidase (HRP) (Cell Signaling technologies; dilution 1:10,000 v/v) for 1 h at room temperature. The blots were developed using a chemiluminescence coupled reaction. The band intensities on the X-ray films were quantitated by using ImageJ software, and normalized.

### CRISPR/Cas9-mediated deletion of Bmi1 in murine pre B-ALL Ba/F3 cells

The CRISPR/Cas9 control plasmid vector (Catalog sc-418922) containing scramble guide RNA (Scr gRNA) and Bmi1 CRISPR/Cas9 knock out plasmid vector (Catalog sc-419338) containing Bmi1 targeting gRNA (Bmi1 gRNA) were purchased from Santa Cruz Biotechnology, Inc. Freshly passaged murine pre B-ALL Ba/F3 cells (2 x 10^6^) were nucleofected with 2 μg of scramble or Bmi1 targeting gRNA containing CRISPR/Cas9 plasmid vector using SG Cell line 4D-Nucleofector X Kit and 100 ml nucleocuvette vessel (Lonza, Catalog V4XC-3012) and nucleofection program CM-147, as per manufacturer’s instruction. Following nucleofection, cell were immediately resuspended in fresh media and transferred to a 12-well non tissue culture plate, and 72 h post nucleofection the EGFP^+^ (nucleofected) cells were sorted and used for western blot (anti−Bmi1 rabbit mAb used at 2.1 nM concentration) and confocal immunofluorescence microscopy (anti−Bmi1 Rabbit mAb at 21.7 nM concentration).

### Transcriptome and bioinformatics analysis

Total RNA was extracted from sorted p190-BCR-ABL^+^ B-cell progenitors (FSC^hi^ EYFP^+^B220^lo^CD19^+^IgM^−^CD43^+^) derived from WT and *Vav3*^−/−^ leukemic primary mice using RNeasy Mini Kit (QIAGEN). RNA quality and concentration were measured by Bioanalyzer 2100 using the RNA 6000 Nano Assay (Agilent Technologies). RNA-seq libraries were prepared using the Illumina TruSeq RNA preparation kit and sequenced on the Illumina HiSeq 2500 using paired-end 75 bp reads. Reads were aligned with TopHat software, using mm10 as the reference genome and mapping reads per kilobase per million mapped reads (RPKM) as output. RPKM were log2-transformed and base-lined to the median expression of the average of each class of samples. RNA samples were processed for RNAseq analyses using RNA-seq protocol from NuGEN and Illumina. The amplified products were sequenced to analyze the gene expression profile. The transcriptome data were further analyzed for differential expression using Altanalyze^76^ and gene-ontology of molecular and biological functions and pathway analyses were performed using ToppGene Suites^77^ and DAVID (Database for Annotation, Visualization and Integrated Discovery, v6.8)^78^.

### Quantitative RT-PCR

Total RNA was extracted from WT, *Vav3*^−/−^, *Rac2*^−/−^ p190-BCR-ABL^+^ B-cell progenitors transduced with empty or Bmi1 or Phlpp2 lentiviral vectors using RNeasy minikit (QIAGEN; catalog 74104) following manufacturer’s instructions, and cDNA was prepared using Taq Man reverse transcription reagent (Applied Biosystems, Life technologies, catalog N8080234). The mRNA expression levels of *Cdkn2a, Cdkn2b, Pax5, Ebf1, Ikzf1*, and *Phlpp2* were analyzed by Q-RT-PCR (qRT-PCR) assay using TaqMan Universal PCR master mix and gene-specific TaqMan primers (Roche Applied Science, Life technologies). The expression level was normalized to the expression of internal control gene *Gapdh*.

### Cleavage under target and release using nuclease sequencing (CUT&RUNseq) assay

The CUT&RUN assays were performed by following method published by Henikoff’s group with minor modifications^79^. Briefly, WT, *Vav3*^*−*/*−*^ p190-BCR-ABL^+^ B-cell progenitors (B220^+/lo^Cd19^+^IgM^-^Cd43^+^) were sorted from the BM (pooled, *n* = 3) of leukemic mice, and CUT&RUN experiment for WT and *Vav3*^*−*/*−*^ leukemic B-cell progenitors were performed in duplicates (*n* = 2). For each CUT&RUN assay, 150,000 cells were taken, washed twice with wash buffer [20 mM HEPES (PH-7.5), 150 mM NaCl, 0.5 mM spermidine] containing protease inhibitor cocktail (Roche, Catalog. 11873580001). Cells were bound to Concavalin A magnetic beads (Bangs Laboratories, Inc. Catalog. BP531) followed by permeabilization and antibody binding (anti-Bmi1, anti-Ring1b, and anti-H2AK119Ub antibodies used at 21.7 nM concentration) in wash buffer containing 0.025% digitonin. The specificity of the anti-Bmi1 antibody was demonstrated in Bmi1-deleted (CRISPR/Cas9) pre-B B-ALL Ba/F3 cells (Supplementary Fig. 6A, B) and antibodies (αRing1b and αH2AK119Ub) had been previously validated for chromatin immunoprecipitation^46^. Antibody bound cells were treated with pA-MNase (700 ng/mL) for 1 h at 4 °C followed by targeted digestion in presence of CaCl2 at 0 °C for 30 min. The digestion reaction was stopped by adding STOP buffer containing 20 mM EDTA and 4mM EGTA. The MNase digested DNA fragments were released at 37 °C, and extracted using QIAGEN MinElute Reaction Cleanup kits (Catalog. 28204). The CUT&RUNseq libraries were prepared by using NEBNext Ultra II DNA library preparation kit for Illumina (Catalog. E7103S) and NEBNext multiplex Oligoes for Illumina (Catalog. E7335S). The libraries were run on Illumina sequencer NextSeq500 with paired end sequencing. The antibodies used for CUT&RUNseq assay were α−H2AK119Ub Ab (Catalog.8240), α−Bmi1 Ab (Catalog.5856), and α−Ring1b (Catalog. 5694) and Isotype control IgG (Catalog. 3900) from Cell Signaling Technologies.

Data analysis was performed in Scientific Data Analyses platform “SciDAP” (https://scidap.com, Datirium, LLC) using “TrimGalore Chip-Seq PE” pipeline. This and other containerized CWL pipelines used in analysis are available at https://github.com/datirium/workflows. Briefly, adapters were trimmed from raw reads with Trim Galore and the reads were aligned to the mm10 reference genome with BowTie^80^. Maximum three mismatches per read were allowed. Only uniquely mapped reads were reported. In the next step, all PCR duplicates were removed by Samtools^81^. For peak calling MACS2^82^ was run with the FDR of 0.05. Data reported include number of peaks called, mean peak size, total reads/pairs in the treatment group, reads/pairs after filtering in treatment, and fraction of reads in peaks (FRIP; Supplementary Tables 1-2). Reported peaks were used in the differential binding analysis and description for specific loci in Supplementary Figs. 10A-B included, in sequence, the integer score of each peak, the fold-change at peak summit and the statistical analysis (FDR) presented as -log10q value at the peak summit. Clustered pairwise correlation matrix of Bmi1, Ring1b, and H2AK119Ub CUT&RUN datasets for WT and *Vav3*^*−*/*−*^, calculated based on the read coverages computed across the entire genome with the bin size of 5 Kb (Pearson’s r test). All calculations were made in deepTools package using multiBamSummary and plotCorrelation commands^83^.

Differentially Bmi1, Ring1b and H2AK119Ub bound sites between WT and *Vav3*^*−*/*−*^ leukemic B-cell progenitors (*n* = 2/group) were identified by using Diffbind- Differential Binding Analysis of ChIP-Seq Peak Data - 1.0.0^84^ *pipeline attached to SciDAP platform*, using “Deseq2” analyses method. Only significant differentially bound sites with *p*-value ≤ 0.05 and with a minimum of 2 fold change (Log_2_Fold Change ≥1 and ≤−1) were reported. Based on this, all differential peaks were divided into two groups: (1) Log_2_FC ≥ 1, (2) Log_2_FC ≤ −1. Each peak group was cleaned from duplicates based on the peak start and end coordinates and centered by peak center. Re-centered peaks were used for generating tag-density plots within 20 kb radius from peak center with “Homer”. For gene TSS-centered tag density plots, each peak was assigned to the nearest gene within 20 kb radius from TSS. The resulted two groups of genes were deduplicated and intersected based on the gene names thus obtaining three groups of genes. Genes from every group were re-centered on the TSS and used for generating tag-density heatmap within 20 kb radius from gene TSS with Homer. The tag density maps were generated using https://software.broadinstitute.org/morpheus/. The representative genome browser map of specific loci was obtained from IGV browser in “SciDAP” platform. The Venn diagram of genes with differential Bmi1, Ring1b and H2AK119Ub binding between WT and *Vav3*^*−*/*−*^ leukemic B-cell progenitors were generated using online tool “Multiple List comparator” (https://www.molbiotools.com/listcompare.php). The significance of overlapped genes and exact test of multi-set intersection were evaluated using tool (https://cran.r-project.org/)^85^. The genes differentially bound in *Vav3*^*−*/*−*^ leukemic B-cell progenitors in comparison to their WT counterparts were subjected to gene ontology analyses (molecular and biological functions and pathway analyses) using ToppGene Suite (https://toppgene.cchmc.org/enrichment.jsp) and confirmed using bioinformatics platform DAVID (Database for Annotation, Visualization and Integrated Discovery, v6.8^78^).

### Whole exome sequencing

For whole exome sequencing, genomic DNA from p190-BCR-ABL-induced leukemic mice [WT (*n* = 4) and *Vav3*^*−*/*−*^ (*n* = 4)] were isolated following lysis of cells in DNA extraction buffer (5 mM EDTA, pH-8; 200 mM NaCl; 100 mM Tris, pH-8; 0.2% SDA, and 20 mg/ml proteinase K), precipitation with 50% isopropanol and washing with 70% ethanol. Following an initial Quality Control evaluation, 50 ng of genomic DNA for each sample was used for NGS library preparation and multiplex exome enrichment (both Twist Biosciences).  Libraries were evaluated for quantity and quality and pooled together for sequencing on an Illumina NovaSeq 6000 instrument. Each sample targeted 30M PE-100 read pairs.

Raw sequencing data was aligned to the mm10 genome with BWA-MEM version 0.7.17^86^ using the non-default parameter “-Y”.  Alignment files were sorted and duplicate reads identified with the bamsormadup program found in the biobambam2 suite of tools (version 2.0.87).  Variants were called with GATK4 v4.1.8.0. The HaplotypeCaller tool was first used to create gvcfs for each sample with parameters “-max-alternate-alleles 3 -ip 100” and the bed file containing capture regions provided by the manufacturer. Finally, variants were genotyped with the GenotypeGVCFs tool.  In order to achieve maximal sensitivity, SNPs were not filtered beyond the default calling thresholds used by GATK.  However, indels were filtered with bcftools v1.10.2 and the expression “TYPE != "snp" && (QD < 2.0 || ReadPosRankSum < −20.0 || FS > 200.0 || SOR > 10.0)”.  Finally, gene annotations and variant consequences were annotated using the Ensembl REST web server. Copy number variation was analyzed using the CNVKit tool^87^ and the UCSC Reference Genome Browser database (http://genome.ucsc.edu). C57Bl/6 murine reference was used for alignment and data was filtered for clinically relevant loci.

### Statistical analysis

Quantitative data is given as mean ± standard deviation (SD). Statistical significance was determined using the unpaired Student-*t* or Anova tests and differences in survival were examined using the log-rank *P* test. A value of *p* < 0.05 was considered to be statistically significant.

### Reporting summary

Further information on research design is available in the Nature Research Reporting Summary linked to this article.

## Supplementary information

Supplementary Information

Description of Additional Supplementary Files

Supplementary Data 1

Supplementary Data 2

Supplementary Data 3

Supplementary Data 4

Supplementary Data 5

Supplementary Data 6

Supplementary Data 7

Supplementary Data 8

Reporting Summary

## Data Availability

The authors declare that all data supporting the findings of this study are available, to the best of our effort, within this manuscript and supplementary information files. Source data are provided with this paper. DNA sequencing data can be accessed at BioProject PRJNA675836. RNA and CUT&RUN sequencing data can be accessed at GEO dataset GSE196378. Source data are provided with this paper.

## Source data

Source Data
