# Supplementary material for: Nuclear Vav3 is required for polycomb repression complex-1 activity in B-cell lymphoblastic leukemogenesis
Source: Nat Commun. 2022 Jun 1;13:3056. doi: 10.1038/s41467-022-30651-7 (PMC9160250; doi:10.1038/s41467-022-30651-7)
Supplement: Supplementary file 1 — Supplementary Information [file 41467_2022_30651_MOESM1_ESM.pdf]

Supplementary Figures

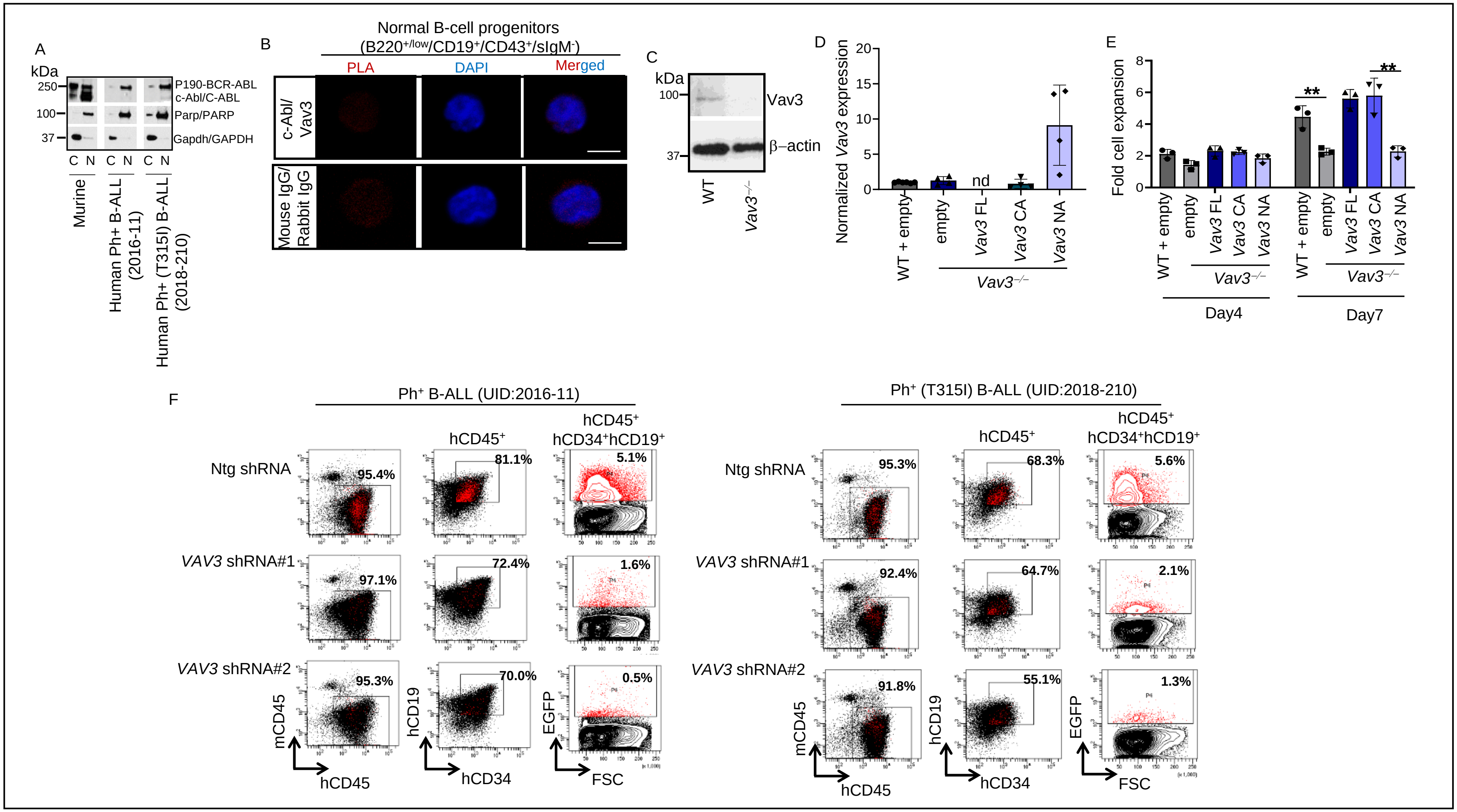

Supplementary Figure 1. VAV3/Vav3 expression and activity are increased in murine and human B-ALL B-cell progenitors.

A) Immunoblot showing predominant nuclear distribution of BCR-ABL in murine and human B-ALL B-cell progenitors. B) Representative confocal images of proximity ligation assay (PLA) between c-ABL and Vav3 in murine B-cell progenitors. C) Western blot for Vav3 in WT and *Vav3*$^{-/-}$ B-cell progenitors. Bar size = 10 μm. D) Q-RT-PCR analyses of Vav3 expression in total RNA isolated from WT (empty vector transduced) and *Vav3*$^{-/-}$ leukemic B-cell progenitors transduced with empty vector, Vav3 FL, Vav3

CA and Vav3NA mutants (n=4 per group). n.d. = not detected after 50 cycles of amplification. E) Expansion of Vav3 mutant and p190 BCR-ABL transduced *Vav3*$^{-/-}$ B-cell progenitors ex vivo (n=3 per group). F) Examples of BM chimera analysis (with percentages of different gated populations) by FACS showing human B-ALL chimera transduced with Ntg or *VAV3* shRNAs (EGFP). Data are presented as mean ± SD in onr to two independent experiments. **, p<0.01.

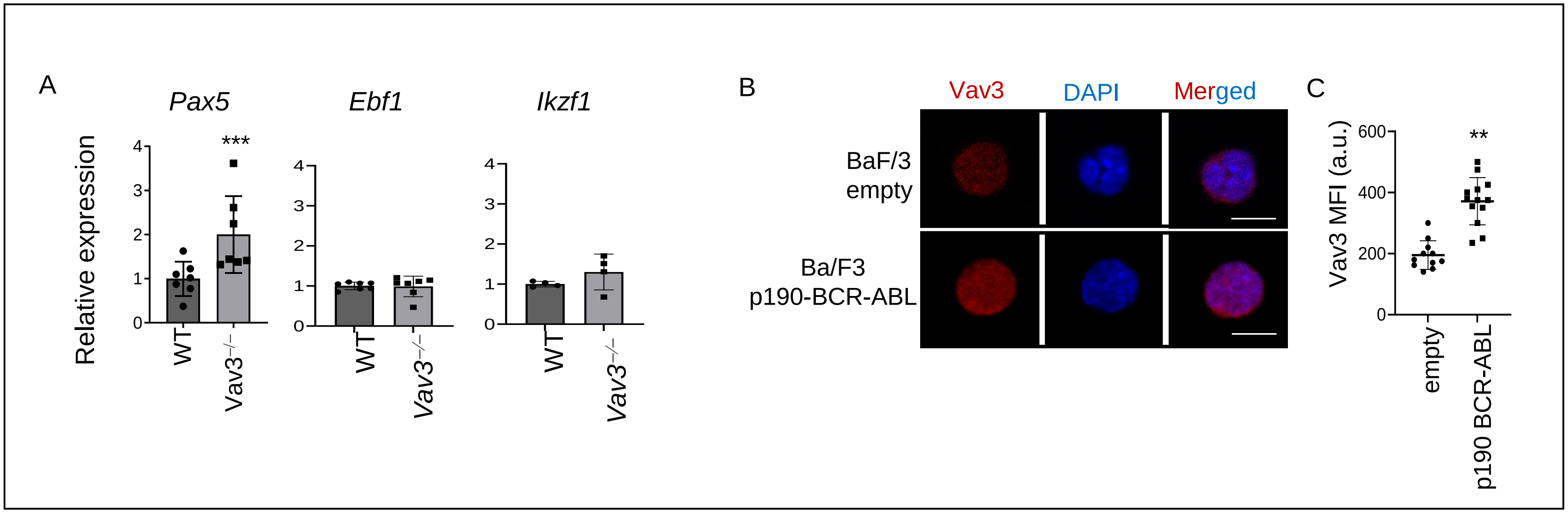

Supplementary Figure 2. Analyses of the expressions of *Pax5, Ebf1* and *Ikzf1* in WT and *Vav3*$^{-/-}$ leukemic B-cell progenitors, and Vav3 expression and distribution in empty vector or p190 BCR-ABL transduced Ba/F3 cells. A) Q-RT-PCR analyses of *Pax5*, *Ebf1* and *Ikzf1* in total RNA isolated from WT and *Vav3*$^{-/-}$ leukemic B-cell progenitors (n=4-7 per group). *Pax5* is upregulated while expression of *Ebf1* and *Ikzf1* remain unchanged in *Vav3*$^{-/-}$ cells. B) Confocal Immunofluorescence images of Vav3 expression and nuclear distribution in mock and p190 BCR-ABL transduced Ba/F3 cells. Bar size = 10 μm. C) Vav3 mean fluorescence intensity

quantification (n=11-13 per group). a.u., arbitrary unit. Data are presented as mean ± SD in two independent experiments. ** p<0.01; *** p < 0.001.

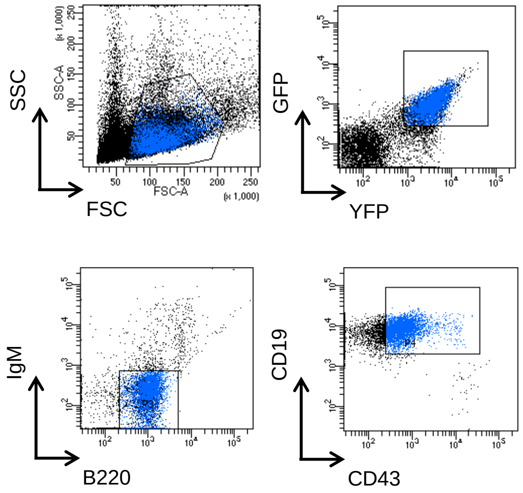

Supplementary Figure 3. Immunophenotypic characterization of murine Ph^+ B-ALL. Immunophenotypic characterization of p190 BCR-ABL^+ B-cell progenitors ($EYFP^+EGFP^+FSC^{hi}B220^{lo}CD19^+IgM^-CD43^+$). Leukemic, moribund *Vav3*$^{-/-}$ chimeric animals have similar immunophenotype although latency to develop the disease is significantly prolonged.

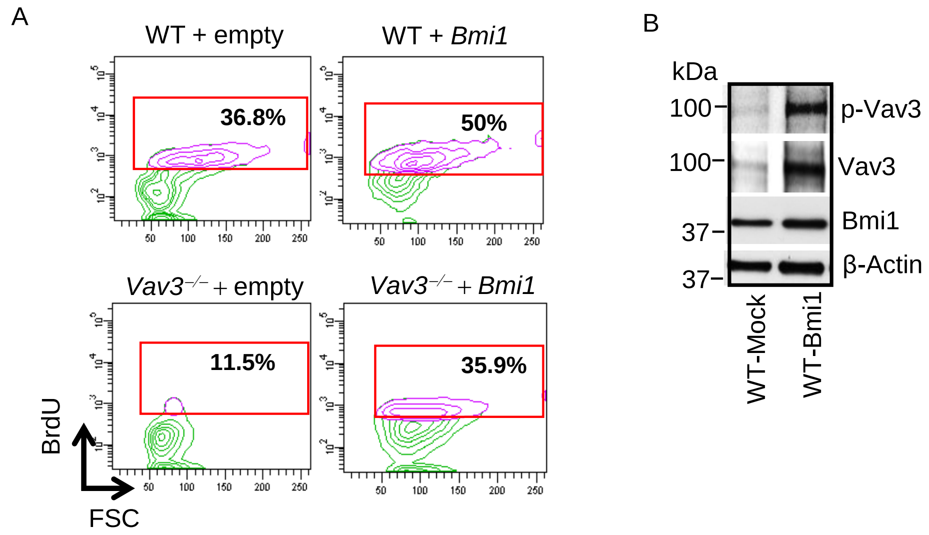

Supplementary Figure 4. Vav3 deficiency attenuates the effect of Bmi1 over-expression on the survival and proliferation of p190 BCR-ABL^{+} B-cell progenitors. A) FACS contour plot analyses of *in vivo* BrDU uptake showing reduced proliferation of Vav3 deficient leukemic B-cell progenitors. Bmi1 overexpression in WT group shows increased proliferation compared with empty vector transduced WT group. Vav3 deficiency attenuates the oncogenic effect of Bmi1 on survival and proliferation. B) Representative immunoblots of Bmi1, Vav3 and p-Vav3 in empty vector or Bmi1 over expression vector transduced leukemic B-cell progenitors. Modestly upregulated expression of Bmi1 leads to increased expression and activation of Vav3. Data are presented as mean ± SD in three independent experiments. **, p<0.01; ***, p<0.001.

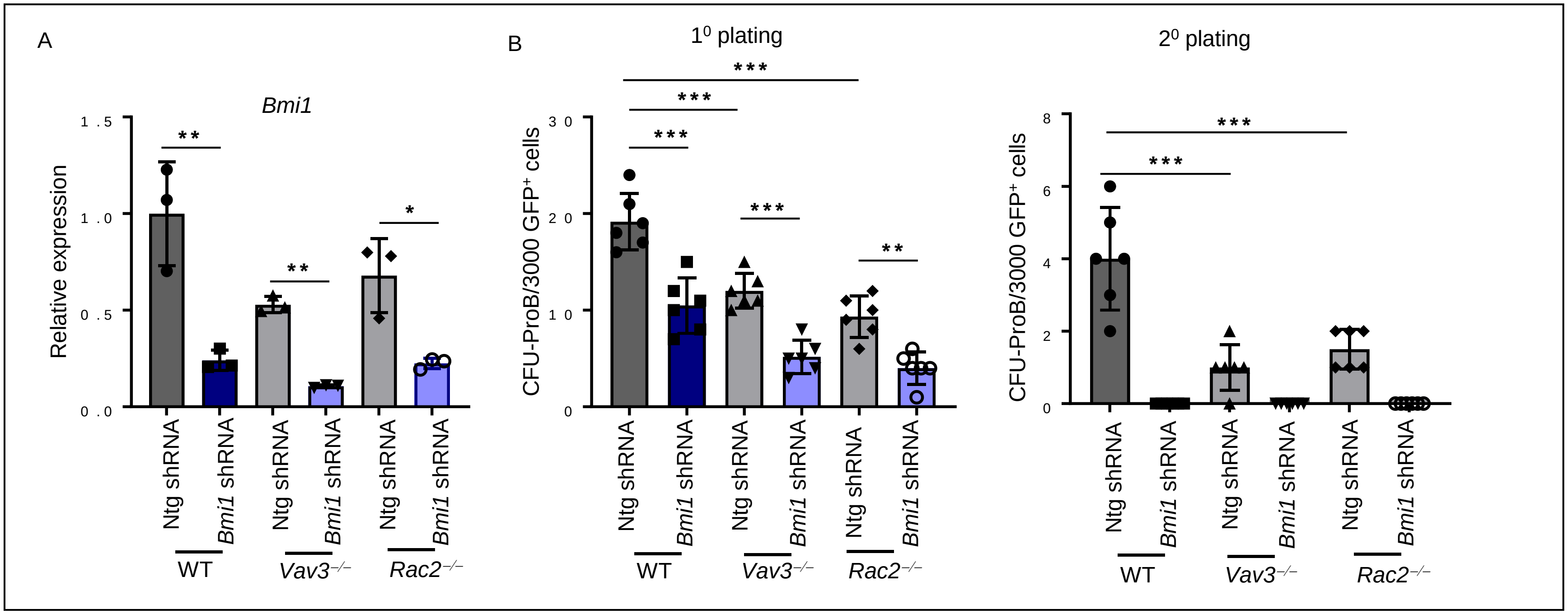

Supplementary Figure 5. shRNA mediated depletion of Bmi1 synergizes with Vav3 and Rac2 deficient leukemic B progenitors replating ability. A) Q-RT-PCR showing depletion of Bmi1 mRNA levels in Bmi1 shRNA transduced WT, *Vav3*$^{-/-}$, *Rac2*$^{-/-}$ leukemic B-cell progenitors (n=3 per group). B) Serial plating of CFU-proB generating ability of Ntg/Bmi1 shRNA transduced WT, *Vav3*$^{-/-}$ and *Rac2*$^{-/-}$ leukemic B-cell progenitors. Vav3 and Rac2 deficiencies led to reduced CFU-proB generating ability of p190-BCR-ABL^{+} leukemic cells. Deficiency of Bmi1 exacerbate the effect of Vav3 and Rac2 deficiencies on CFU generation in both primary and secondary plating (n=3 per group). No colonies were observed in Bmi1 shRNA transduced group in secondary plating (represented as zero values). Data are presented as mean ± SD of one to three independent experiments. *, p<0.05; **, p<0.01.

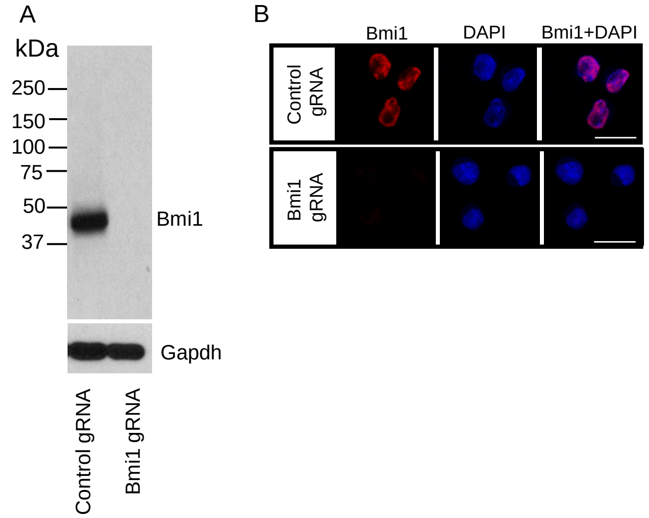

Supplementary Figure 6. CRISPR/Cas9 mediated deletion of Bmi1 in murine pre B-ALL Ba/F3 cells. A-B) Western blot (A) and confocal microscopic images (B) of Bmi1 in Ba/F3 cells nucleofected with CRiSPR/Cas9 vector carrying scramble guide RNA or Bmi1 targeting guide RNA. Cells transfected with CRiSPR/Cas9 vector carrying Scramble gRNA shows nuclear Bmi1 expression, and Bmi1 targeting vector nucleofected cells shows Bmi1 knockout. Data are representative of one independent experiment, with two replicates. Bar size = 20 μm.

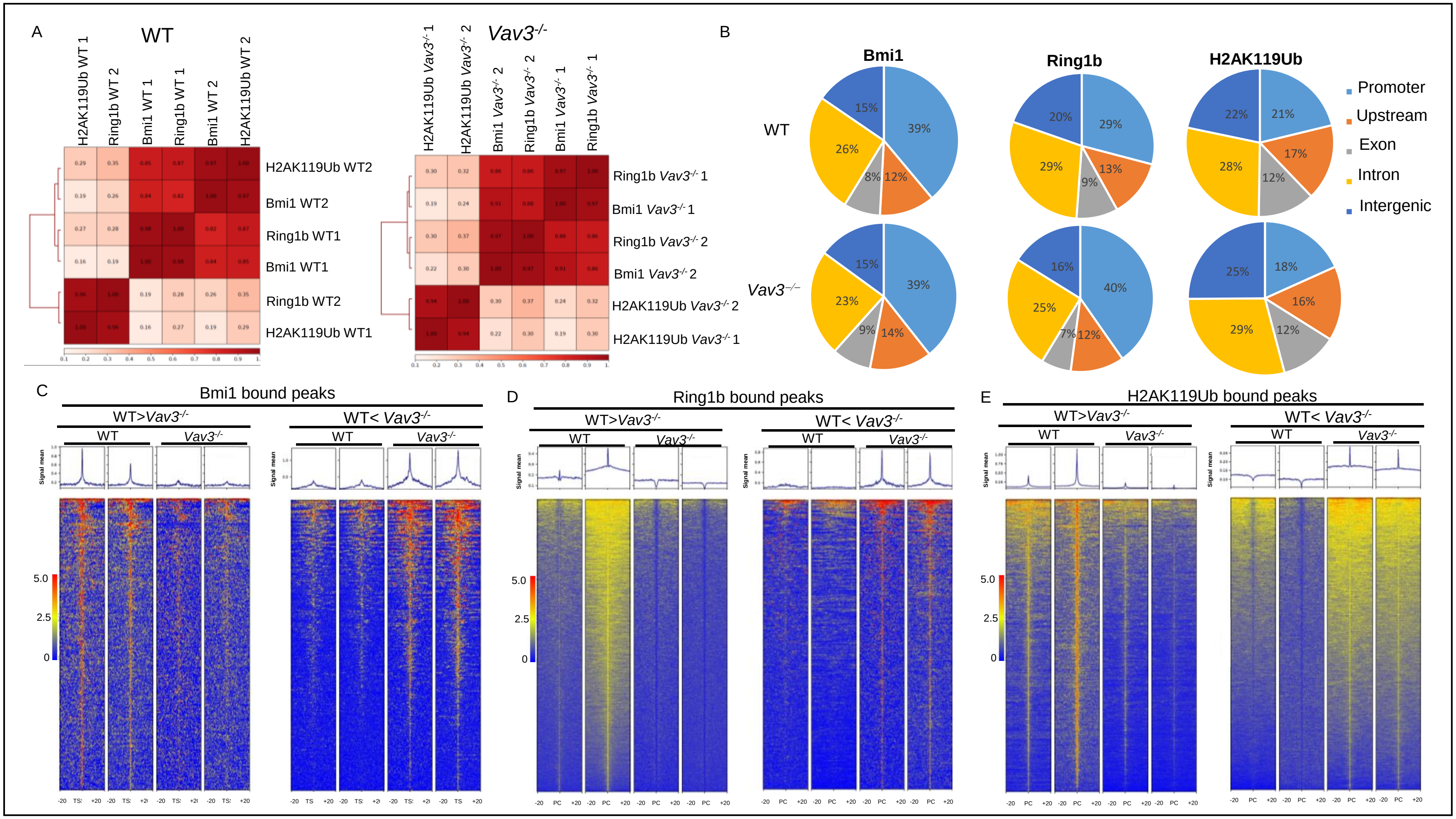

Supplementary Figure 7. Whole genome wide analyses of Bmi1, Ring1b and H2AK119Ub CUT&RUNseq peak distribution. A) Clustered pairwise correlation matrix of Bmi1, Ring1b, and H2AK119Ub CUT&RUN datasets for WT and *Vav3*$^{-/-}$ leukemic B-cell progenitors, calculated based on the read coverages computed across the entire genome with the bin size of 5 Kb using Pearson correlation method. Last digit depicts the number of independent experiment. B) Whole genome distribution of Bmi1, Ring1b and H2AK119Ub binding peaks to promoter (±1 Kb from TSS), upstream (-20Kb and -1Kb from TSS), exon, intron and intergenic regions in WT and *Vav3*$^{-/-}$ leukemic B-cell progenitors. (C-E) Density maps and average tag density profiles around ± 20Kb regions of peak

centers (PC) for loci with decreased (left panels) or increased (right panels) Bmi1, Ring1b or H2AK119b antibody binding in *Vav3*$^{-/-}$ leukemic B-cell progenitors, by duplicate. (C) Bmi1. (D) Ring1b. (E) H2AK119Ub. Data derived from one replicate from two independent experiments.

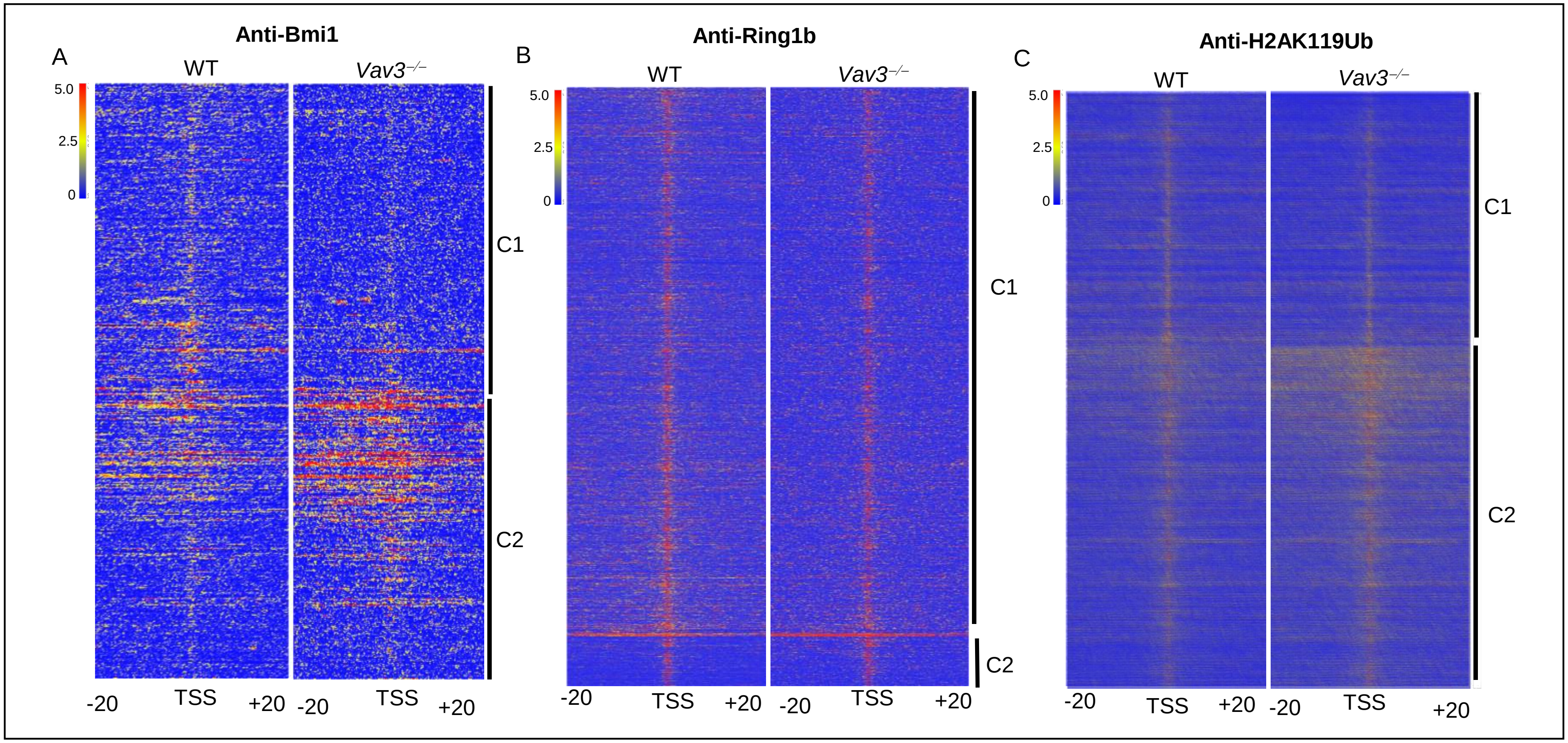

Supplementary Figure 8. Density maps of differential binding (around ± 20Kb regions of TSS) of loci by Bmi1 (A), Ring1b (B) and H2AK119Ub (C) in WT and *Vav3*$^{-/-}$ leukemic B-cell progenitors. We performed "Diffbind" analyses of 2 replicates in WT and *Vav3*$^{-/-}$ leukemic B-cell progenitors, the differential bound peaks (≥2folds enriched and p value ≤0.05) were considered and assigned to nearest genes, and 1 representative replicate from each group is presented here. C1 represents the cluster 1 including genes that

had lower binding for each of the three antibodies. C2 represents the cluster including genes that had higher binding for each of the three antibodies.

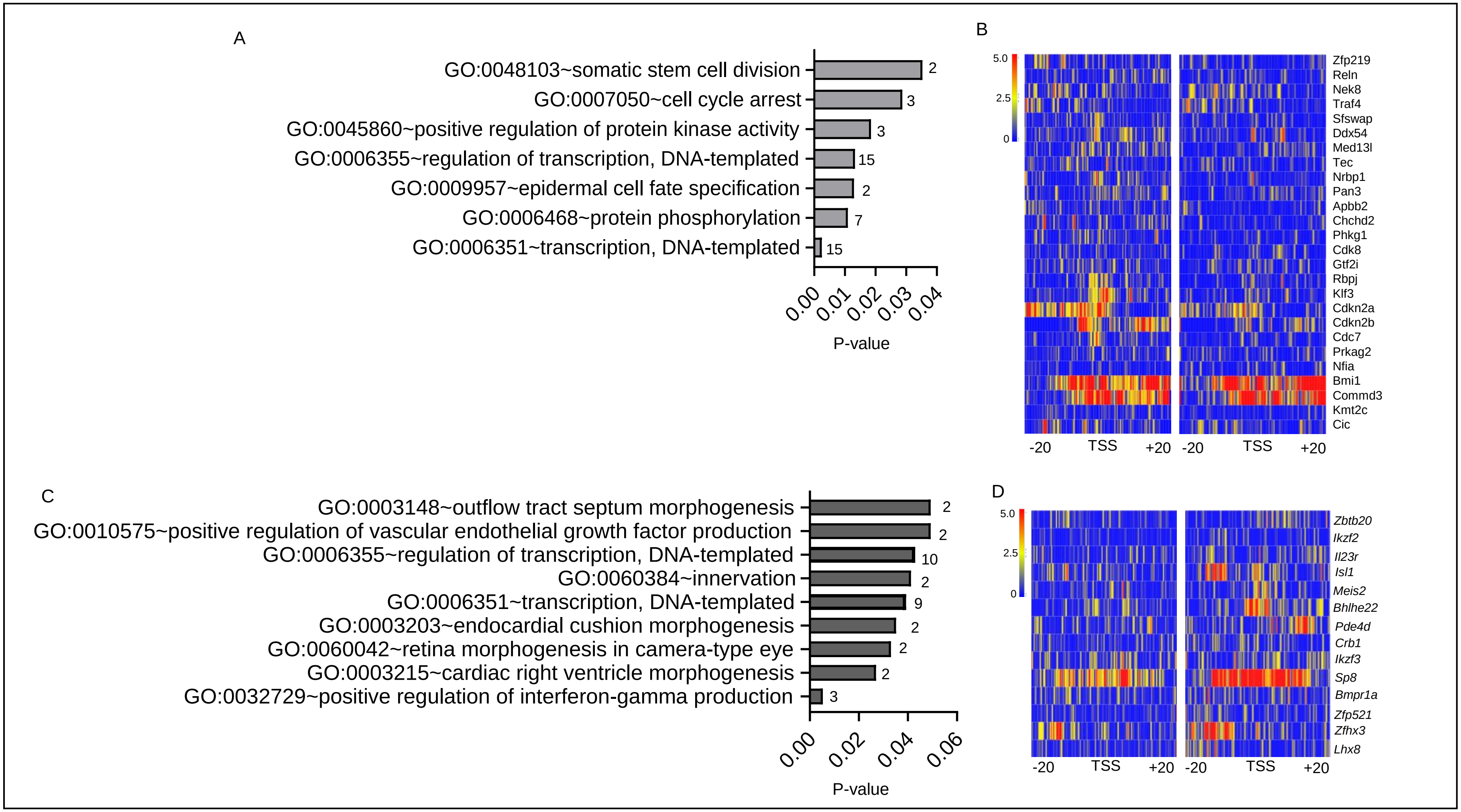

Supplementary Figure 9. Gene ontology analysis of integrated CUT&RUNseq analyses. A) GO term biological process analyses of 75 common genes with reduced Bmi1, Ring1b and H2AK119Ub occupancy using bioinformatics tool DAVID. B) Density map (± 20Kb regions of TSS) of loci associated with GO in A. C) GO term biological process analyses of 50 common genes with increased Bmi1, Ring1b and H2AK119Ub occupancy using DAVID GO analysis. D) Density map (± 20Kb regions of TSS) of loci associated with GO in C.

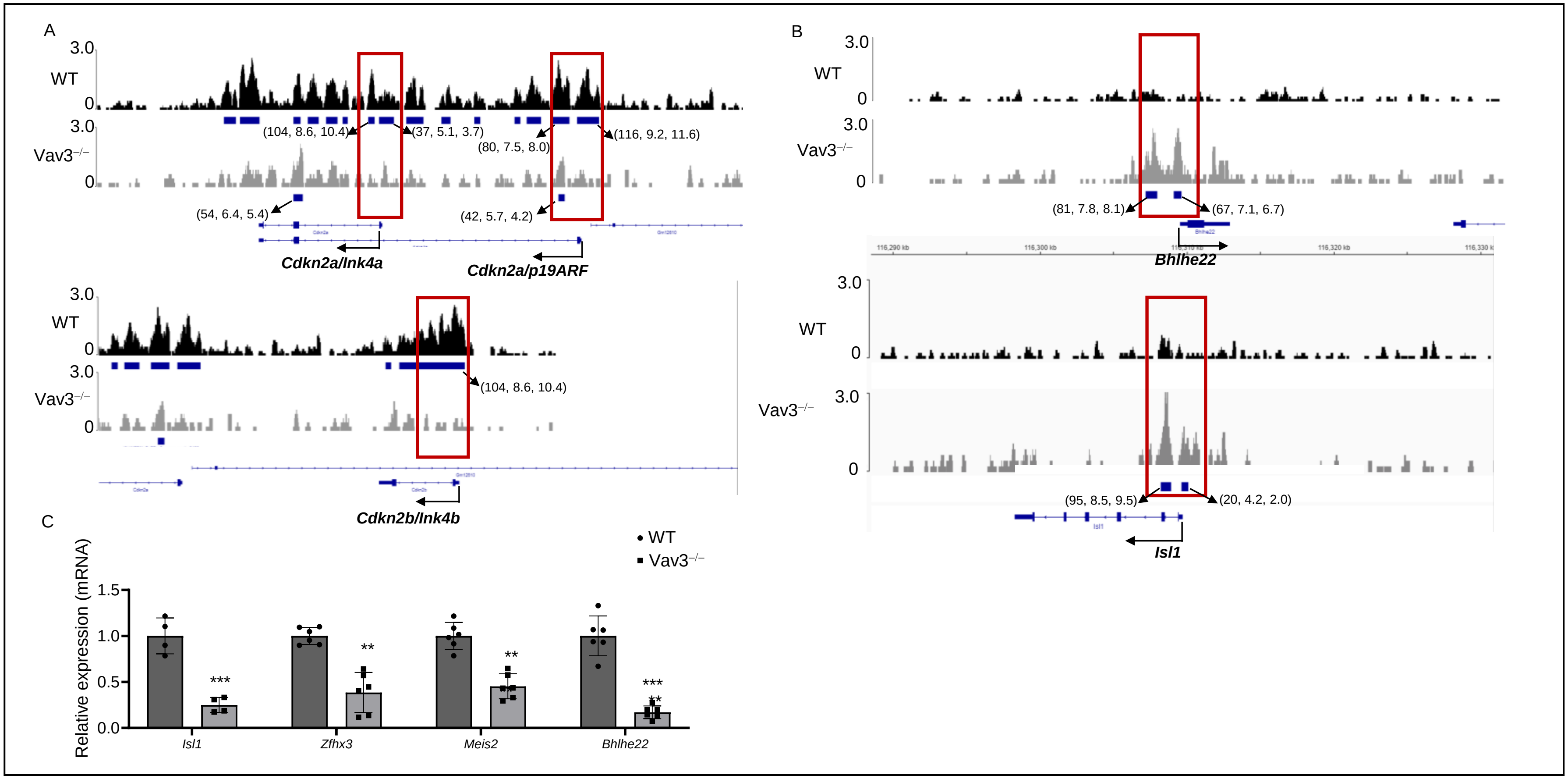

Supplementary Figure 10. Representative genome browser map of Bmi1 association at loci involved in regulation of proliferation and regulation of G0/G1 transition (Figure 4B) and regulation of polII dependent transcription (Figure 4E). A) Genome browser map of Bmi1 at *Cdkn2a and Cdkn2b*. The number as well as intensity of significantly enriched peaks is reduced in *Vav3*$^{-/-}$ leukemic B-cell progenitors. B) Genome browser map of Bmi1 at *Pbx1 and Bhlhe22* loci. C) Q-RT-PCR analyses of the expression of *Bhlhe22, Pbx1, Zfp521, Zfhx3, Ikzf2, Ikzf3, Pde4d and Pde10a* genes in WT and *Vav3*$^{-/-}$ leukemic B-cell progenitors. The integer score, peak intensity at the summit and statistical FDR probability expressed as –log10q value of the significantly enriched peaks (marked by solid bar below) are indicated sequentially within parentheses and separated by commas under the

specific differentially enriched peaks (n=4-6 per group). Data in graphs are presented as mean ± SD of two independent experiments. * $p < 0.05$; ** $p < 0.01$.

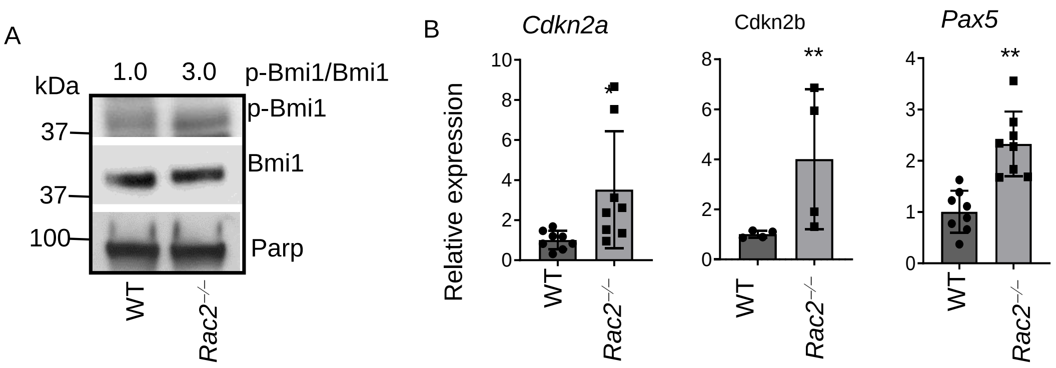

Supplementary Figure 11. Rac2 deletion leads to increased Bmi1 phosphorylation and increased expression of PRC1.4 downstream targets *Cdkn2a, Cdkn2b* and *Pax5*. A) Representative western blots of p-Bmi1, Bmi1 and Parp in the nuclear fraction of WT and *Rac2*$^{-/-}$ leukemic B-cell progenitors. Rac2 deficiency led to increased Bmi1 phosphorylation. B) Q-RT-PCR analyses of total RNA isolated from WT and *Rac2*$^{-/-}$ leukemic B-cell progenitors (n=4-8 per group). The expression of *Cdkn2a, Cdkn2b* and *Pax5* is increased in *Rac2*$^{-/-}$ leukemic cells. Data in graphs are presented as mean ± SD of 3 independent experiments. *, $p<0.05$; **, $p<0.01$.

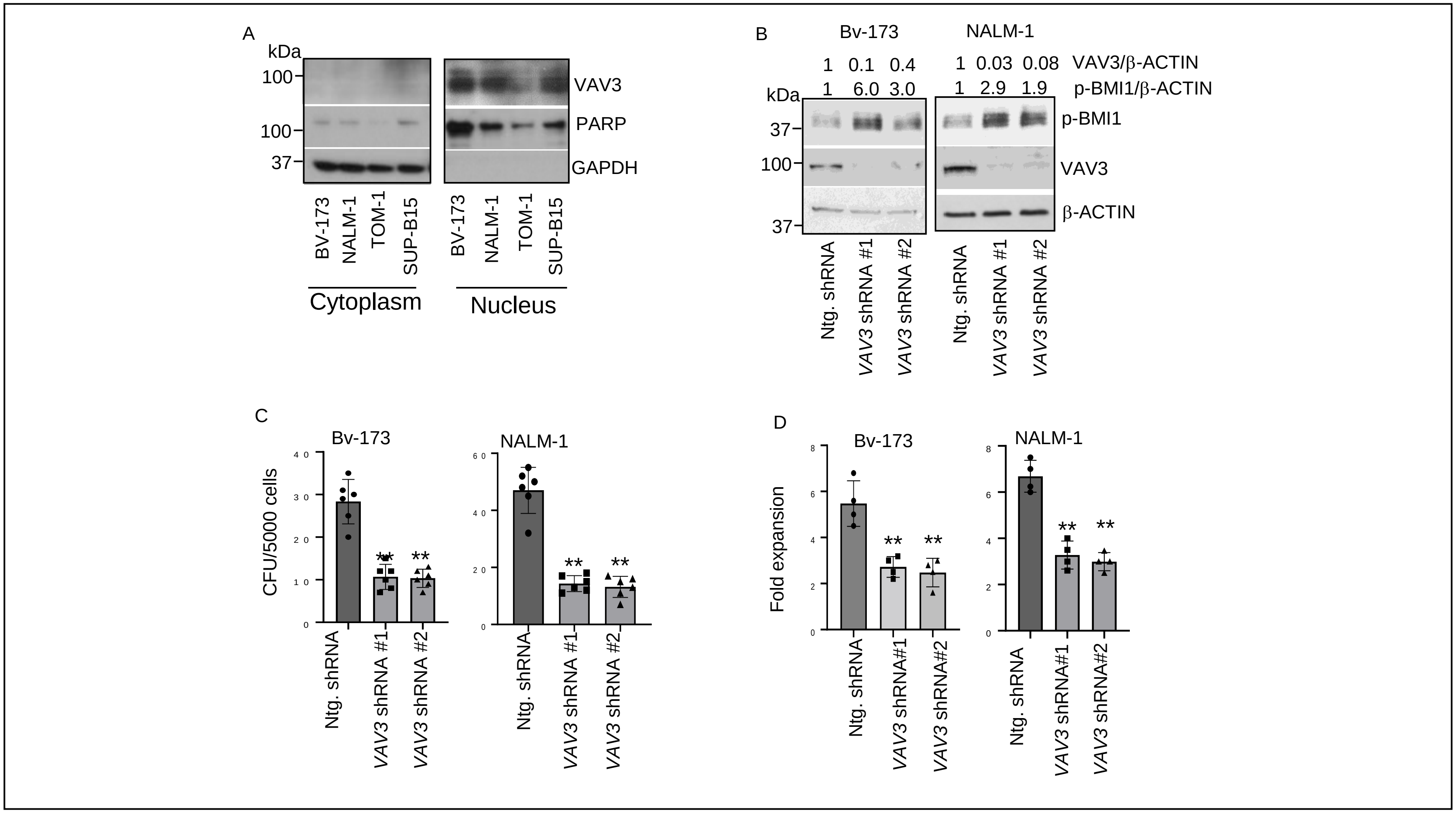

Supplementary Figure 12. VAV3 downregulation leads to increased BMI1 phosphorylation in human B-cell precursor leukemic cell lines. A) Representative examples of western blots of VAV3, PARP and GAPDH in the cytoplasm and nucleus of human B-cell precursor leukemic cell lines Bv-173, NALM-1, TOM-1 and SUP-B15. B) Representative examples of western blots of p-BMI1, VAV3 and β-ACTIN in VAV3 shRNA transduced Bv-173 and NALM-1 cells. C) Frequency of CFU-blast in non-targeted (Ntg) shRNA and VAV3 shRNA transduced Bv-173 and NALM-1 cells (n=3 per group). D) Expansion of Ntg shRNA and VAV3 shRNA

transduced human B-precursor leukemia cell lines Bv-173 and NALM-1 growing in liquid culture (n=3 per group). Data are presented as mean ± SD of one to three independent experiments. *, p<0.05; **, p<0.01.

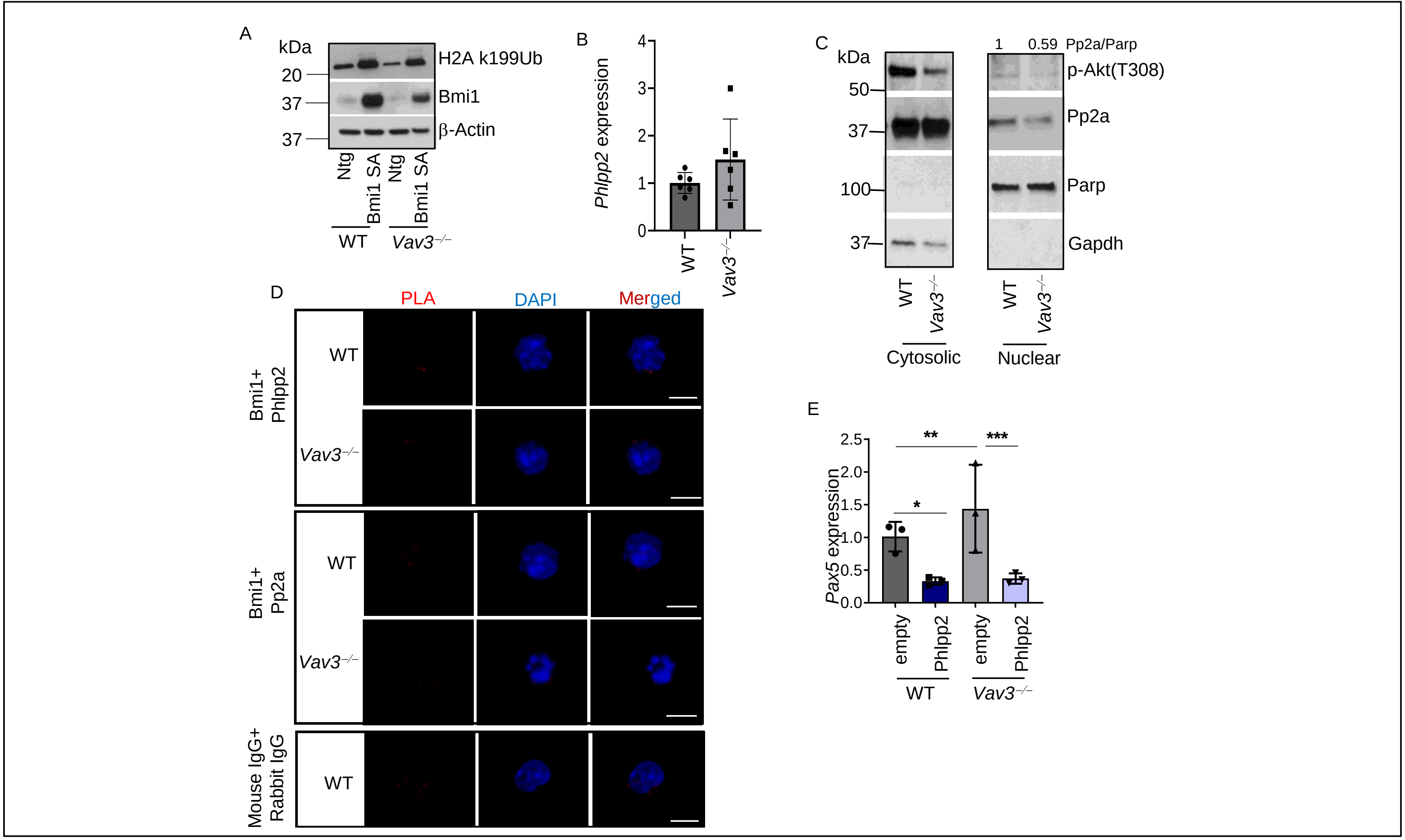

Supplementary Figure 13. Phlpp2/Akt-S314 axis, but not Pp2a/Akt-T308, is modified in *Vav3*$^{-/-}$ B-cell progenitors. A) Western blots of Bmi1, H2AK119Ub and β-Actin in empty or Bmi1S314A lentiviral vector transduced WT and *Vav3*$^{-/-}$ leukemic B-cell

progenitors. B) Q-RT-PCR analyses of *Phlpp2* mRNA expression in WT and *Vav3*$^{-/-}$ B-cell progenitors (n=6 per group). C) Western blots of Pp2a, p-Akt (T308), Parp and Gapdh in the cytosolic and nuclear fraction of WT and *Vav3*$^{-/-}$ B-cell progenitors. D) Confocal immunofluorescence images PLA between Bmi1 and Phlpp2, and Bmi1 and PP2A. E) Q-RT-PCR analyses of *Pax5* expression in empty or Phlpp2 transduced WT and *Vav3*$^{-/-}$ leukemic B cell progenitors (n=3 per group). Bar size = 10 μm. Data are presented as mean ± SD of one to three independent experiments. *, p<0.05; **, p<0.01; ***, p<0.001.

Supplementary Data

Supplementary Data 1. List of differentially regulated genes with statistically significant difference between WT and *Vav3*$^{-/-}$ B-cell progenitors

Supplementary Data 2. Gene-ontology (molecular functions, biological function and pathway) analyses of all the differentially expressed genes in Vav3 deficient p190 BCR-ABL^{+} B-cell progenitors.

Supplementary Data 3. Exome sequencing and evaluation of mutations and/or deletion in genes known to be mutated and/or deleted in B-ALL.

Supplementary data 4. Differentially bound peaks identified by Diffbind analyses of Bmi1 CUT&RUNseq data.

Supplementary Data 5. Differentially bound peaks identified by Diffbind analyses of Ring1b CUT&RUNseq data.

Supplementary Data 6. Differentially bound peaks identified by Diffbind analyses of H2AK119Ub CUT&RUNseq data.

Supplementary Data 7. Overlapping genes with decreased Bmi1, Ring1b and H2AK119Ub occupancy in *Vav3*$^{-/-}$ leukemic B-cell progenitors.

Supplementary Data 8. Overlapping genes with increased Bmi1, Ring1b and H2AK119Ub occupancy in *Vav3*$^{-/-}$ leukemic B-cell progenitors.
