# Supplementary material for: Nuclear Vav3 is required for polycomb repression complex-1 activity in B-cell lymphoblastic leukemogenesis
Source: Nat Commun. 2022 Jun 1;13:3056. doi: 10.1038/s41467-022-30651-7 (PMC9160250; doi:10.1038/s41467-022-30651-7)
Supplement: Supplementary file 2 — Description of Additional Supplementary Files [file 41467_2022_30651_MOESM2_ESM.pdf]

Description of Additional Supplementary Files

File Name: Supplementary Data 1
Description: List of differentially regulated genes with statistically significant difference between WT and *Vav3–/–* B-cell progenitors

File Name: Supplementary Data 2
Description: Gene-ontology (molecular functions, biological function and pathway) analyses of all the differentially expressed genes in Vav3 deficient p190 BCR-ABL+ B-cell progenitors.

File Name: Supplementary Data 3
Description: Exome sequencing and evaluation of mutations and/or deletion in genes known to be mutated and/or deleted in B-ALL.

File Name: Supplementary Data 4
Description: Differentially bound peaks identified by Diffbind analyses of Bmi1 CUT&RUNseq data.

File Name: Supplementary Data 5
Description: Differentially bound peaks identified by Diffbind analyses of Ring1b CUT&RUNseq data

File Name: Supplementary Data 6
Description: Differentially bound peaks identified by Diffbind analyses of H2AK119Ub CUT&RUNseq data.

File Name: Supplementary Data 7
Description: Overlapping genes with decreased Bmi1, Ring1b and H2AK119Ub occupancy in *Vav3−⁄−* leukemic B-cell progenitors.

File Name: Supplementary Data 8
Description: Overlapping genes with increased Bmi1, Ring1b and H2AK119Ub occupancy in *Vav3−⁄−* leukemic B-cell progenitors.
