# Supplementary material for: Nuclear Vav3 is required for polycomb repression complex-1 activity in B-cell lymphoblastic leukemogenesis
Source: Nat Commun. 2022 Jun 1;13:3056. doi: 10.1038/s41467-022-30651-7 (PMC9160250; doi:10.1038/s41467-022-30651-7)
Supplement: Supplementary file 11 — Reporting Summary [file 41467_2022_30651_MOESM11_ESM.pdf]

nature portfolio

Corresponding author(s): Jose A. Cancelas, MD, PhD
Ramesh C. Nayak, PhD

Last updated by author(s): May 8, 2022

Reporting Summary

Nature Portfolio wishes to improve the reproducibility of the work that we publish. This form provides structure for consistency and transparency in reporting. For further information on Nature Portfolio policies, see our Editorial Policies and the Editorial Policy Checklist.

Statistics

For all statistical analyses, confirm that the following items are present in the figure legend, table legend, main text, or Methods section.

n/a	Confirmed	
☐	☒	The exact sample size (*n*) for each experimental group/condition, given as a discrete number and unit of measurement
☐	☒	A statement on whether measurements were taken from distinct samples or whether the same sample was measured repeatedly
☐	☒	The statistical test(s) used AND whether they are one- or two-sided<br>*Only common tests should be described solely by name; describe more complex techniques in the Methods section.*
☒	☐	A description of all covariates tested
☒	☐	A description of any assumptions or corrections, such as tests of normality and adjustment for multiple comparisons
☒	☐	A full description of the statistical parameters including central tendency (e.g. means) or other basic estimates (e.g. regression coefficient) AND variation (e.g. standard deviation) or associated estimates of uncertainty (e.g. confidence intervals)
☐	☒	For null hypothesis testing, the test statistic (e.g. *F*, *t*, *r*) with confidence intervals, effect sizes, degrees of freedom and *P* value noted<br>*Give P values as exact values whenever suitable.*
☒	☐	For Bayesian analysis, information on the choice of priors and Markov chain Monte Carlo settings
☒	☐	For hierarchical and complex designs, identification of the appropriate level for tests and full reporting of outcomes
☒	☐	Estimates of effect sizes (e.g. Cohen's *d*, Pearson's *r*), indicating how they were calculated

Our web collection on statistics for biologists contains articles on many of the points above.

Software and code

Policy information about availability of computer code

Data collection

BD FACS Diva 9.0 or higher version was used for flow cytometry data collection. Confocal microscopy images were acquired using Zeiss Zen 2.6 image acquisition software. DNA libraries were sequenced on the Illumina NextSeq500 or NovaSeq6000 instruments. For RNAseq HiSeq2500 platform was used. Reads were aligned with TopHat software, using mm10 as the reference genome and Reads per Kilobase of Transcript per Million mapped reads (RPKM) as output.

Data analysis

We used SciDAP (Datirium, LLC) for CUT&RUNseq data analyses. Data analysis was performed in Scientific Data Analyses platform "SciDAP" (https://scidap.com, Datirium, LLC) using "TrimGalore Chip-Seq PE" pipeline. This and other containerized CWL pipelines used in analysis are available at https://github.com/datirium/workflows. Briefly, adapters were trimmed from raw reads with Trim Galore and the reads were aligned to the mm10 reference genome with BowTie78. Maximum 3 mismatches per read were allowed. Only uniquely mapped reads were reported. In the next step, all PCR duplicates were removed by Samtools79. For peak calling MACS280 was run with the FDR of 0.05. Data reported include number of peaks called, mean peak size, total reads/pairs in the treatment group, reads/pairs after filtering in treatment and fraction of reads in peaks (FRIP). Reported peaks were used in the differential binding analysis and description for specific loci, in sequence, the integer score of each peak, the fold-change at peak summit and the statistical analysis (FDR) presented as -log10q value at the peak summit. Differentially Bmi1, Ring1b and H2AK119Ub bound sites between WT and Vav3 deficient leukemic B-cell progenitors (n=2/group) were identified by using Diffbind (Differential Binding Analysis of ChIP-Seq Peak Data - 1.0.081 pipeline attached to SciDAP platform), using "Deseq2" analyses method. Only significant differentially bound sites with p-value ≤ 0.05 and with a minimum of 2 fold change (Log2Fold Change ≥ 1 and ≤ -1) were reported. Based on this, all differential peaks were divided into two groups: 1) Log2FC ≥ 1, 2) Log2FC ≤ -1. Each peak group was cleaned from duplicates based on the peak start and end coordinates and centered by peak center. Re-centered peaks were used for generating tag-density plots within 20kb radius from peak center with "Homer". For gene TSS-centered tag density plots, each peak was assigned to the nearest gene within 20kb radius from TSS. The resulted two groups of genes were de-duplicated and intersected based on the gene names thus obtaining three groups of genes. Genes from every group were recentered on the TSS and used for generating tag-density heatmap within 20kb radius from gene TSS with Homer. The tag density maps were generated using https://software.broadinstitute.org/morpheus/. The representative genome browser map of specific loci was obtained from IGV browser in "SciDAP" platform. The Venn diagram of genes with differential Bmi1, Ring1b and H2AK119Ub binding between WT and Vav3 deficient leukemic B-cell

progenitors were generated using online tool “Multiple List comparator” (https://www.molbiotools.com/listcompare.php). The significance of overlapped genes and exact test of multi-set intersection were evaluated using tool (https://cran.r-project.org/), as described previously. The genes differentially bound in Vav3 deficient leukemic B-cell progenitors in comparison to their WT counterparts were subjected to gene ontology analyses (molecular and biological functions and pathway analyses) using ToppGene Suite (https://toppgene.cchmc.org/enrichment.jsp). For RNA seq analyses, the transcriptome data were further analyzed for differential expression using Altanalyze software and gene-ontology of molecular and biological functions and pathway analyses was performed using ToppGene Suites and DAVID (Database for Annotation, Visualization and Integrated Discovery, v6.8). For whole exome sequencing, raw sequencing data was aligned to the mm10 genome with BWA-MEM version 0.7.1783 using the non-default parameter “-Y”. Alignment files were sorted and duplicate reads identified with the bamsormadup program found in the biobambam2 suite of tools (version 2.0.87). Variants were called with GATK4 v4.1.8.0. The HaplotypeCaller tool was first used to create gvcfs for each sample with parameters “-max-alternate-alleles 3 -ip 100” and the bed file containing capture regions provided by the manufacturer. Finally, variants were genotyped with the GenotypeGVCFs tool. In order to achieve maximal sensitivity, SNPs were not filtered beyond the default calling thresholds used by GATK. However, indels were filtered with bcftools v1.10.2 and the expression “TYPE != "snp" && (QD < 2.0 || ReadPosRankSum < -20.0 || FS > 200.0 || SOR > 10.0)”. Finally, gene annotations and variant consequences were annotated using the Ensembl REST web server. Copy number variation was analyzed using the CNVKit tool84 and the UCSC Reference Genome Browser database (http://genome.ucsc.edu). C57Bl/6 murine reference was used for alignment and data was filtered for clinically relevant lociGraphpad Prism 9, Microsoft Excel, Integrative Genomics Viewer (v. 2.8.9) were used for data analyses and presentation.

For manuscripts utilizing custom algorithms or software that are central to the research but not yet described in published literature, software must be made available to editors and reviewers. We strongly encourage code deposition in a community repository (e.g. GitHub). See the Nature Portfolio guidelines for submitting code & software for further information.

Data

Policy information about availability of data

All manuscripts must include a data availability statement. This statement should provide the following information, where applicable:

- Accession codes, unique identifiers, or web links for publicly available datasets
- A description of any restrictions on data availability
- For clinical datasets or third party data, please ensure that the statement adheres to our policy

The authors declare that all data supporting the findings of this study are available, to the best of our effort, within this manuscript and supplementary information files. Raw data that support the findings of this study are available on request from the corresponding authors (J.A.C. and R.C.N.). DNA sequencing data can be accessed at BioProject PRJNA675836 (HYPERLINK "https://nam11.safelinks.protection.outlook.com/?url=https%3A%2F%2Fwww.ncbi.nlm.nih.gov%2Fbioproject%2FPRJNA675836%2F&data=04%7C01%7CCANCELJE%40UCMAIL.UC.EDU%7C2f37fb6bba32436e2b4e08d9f2f7a8cc%7Cf5222e6c5fc648eb8f0373db18203b63%7C1%7C0%7C637807967768972418%7CUnknown%7CTWFpbGZsb3d8eyJWIjoiMC4wLjAwMDAiLCJQIjoiV2luMzIiLCJBTiI6Ik1haWwiLCJXVCI6Mn0%3D%7C3000&sdata=Jd78YiEPt5LqcMk%2F6XHaYezDKUKF9rjViMQIkRc69%2FE%3D&reserved=0" https://www.ncbi.nlm.nih.gov/bioproject/PRJNA675836/). RNA and CUT&RUN sequencing data can be accessed at GEO dataset GSE196378 (https://www.ncbi.nlm.nih.gov/geo/query/acc.cgi?acc=GSE196378).

Field-specific reporting

Please select the one below that is the best fit for your research. If you are not sure, read the appropriate sections before making your selection.

☒ Life sciences ☐ Behavioural & social sciences ☐ Ecological, evolutionary & environmental sciences

For a reference copy of the document with all sections, see nature.com/documents/nr-reporting-summary-flat.pdf

Life sciences study design

All studies must disclose on these points even when the disclosure is negative.

Sample size	All animal experiments have been planned in an effort to provide 60-80% power for a target effect size of 1.2-1.5 (effect size = [mean difference]/SD). A number of 10 mice per group and experimental replicate have been found sufficient for these experiments. The power can be as small as 46% for an effect size of 1, but effects such as this or smaller magnitude were considered only marginally interesting. Differences between two groups are assessed by an unpaired two-tailed Student t-test. Data involving more than two groups are assessed by one-way analysis of variance with Bonferroni correction.
Data exclusions	No data was excluded
Replication	As noted in the main text, figure legends and methods section, the findings in replicates in each experiment presented in the manuscript generated consistent reproducible data.
Randomization	Human specimens were randomly obtained from the CCHMC repository, without any pre-selection beyond being BCR-ABL+, mutant BCR-ABL or Ph-like B-ALL.
Blinding	For confocal image acquisition of immunofluorescence and proximity ligation assay experiments, blinding was applied.

Reporting for specific materials, systems and methods

We require information from authors about some types of materials, experimental systems and methods used in many studies. Here, indicate whether each material, system or method listed is relevant to your study. If you are not sure if a list item applies to your research, read the appropriate section before selecting a response.

Materials & experimental systems

n/a	Involved in the study
☐	☒ Antibodies
☐	☒ Eukaryotic cell lines
☒	☐ Palaeontology and archaeology
☐	☒ Animals and other organisms
☐	☒ Human research participants
☒	☐ Clinical data
☒	☐ Dual use research of concern

Methods

n/a	Involved in the study
☐	☒ ChIP-seq
☐	☒ Flow cytometry
☒	☐ MRI-based neuroimaging

Antibodies

Antibodies used	Details of antibodies used in the study are provided in the Materials and Methods section
Validation	Antibodies were used as per manufacturer's instruction, and details are found in the Materials and Methods section

Eukaryotic cell lines

Policy information about cell lines

Cell line source(s)	Human leukemic cell lines Bv-173, NALM-1, TOM-1, SUP-B15 were obtained from the German Collection of Microorganisms and Cell Cultures (DSMZ; Bv-173, NALM-1 and TOM-1) and American Tissue Culture Collection (ATCC; SUP-B15).
Authentication	The cell lines obtained from DSMZ or ATCC were used within 5 passages. Cells lines were not authenticated in our lab beyond the provider's certification.
Mycoplasma contamination	Mycoplasma sp. negative
Commonly misidentified lines (See ICLAC register)	Commonly misidentified cell lines were not used

Animals and other organisms

Policy information about studies involving animals; ARRIVE guidelines recommended for reporting animal research

Laboratory animals	The generation of Vav3-deficient (Vav3-/-) mice HYPERLINK \l "71 and Rac2-deficient (Rac2-/-) mice HYPERLINK \l "72 have been described previously. All mutant mice were backcrossed > 10 generations into C57Bl/10 or C57Bl/6 mice, respectively. To avoid possible interference with androgen signaling, 6- to 8-week-old female wild-type (WT) C57Bl/10 and C57Bl/6 mice were obtained commercially (Jackson Laboratory, Ban Harbor, ME and Harlan Laboratories, Indianapolis, IN, respectively) and used as donors and/or recipients for transduction/transplantation models. All mouse strains were maintained at an Association for Assessment and Accreditation of Laboratory Animal Care accredited, specific-pathogen–free animal facility at Cincinnati Children’s Research Foundation, Cincinnati, under an Institutional Animal Care and Use Committee approved protocol. The transgenic mice used in the study were between 6 and 12 week of age at the time of experimentation.
Wild animals	No wild animals were used in this study
Field-collected samples	No field-collected samples were used in this study
Ethics oversight	Laboratory animals were maintained by an Association for Assessment and Accreditation of Laboratory Animal Care accredited facility (at Cincinnati Children's Hospital Medical Center, CCHMC). Specific-pathogen free animal facility was used, under protocol IACUC 2020-0021

Note that full information on the approval of the study protocol must also be provided in the manuscript.

Human research participants

Policy information about studies involving human research participants

Population characteristics	B-ALL patient samples were obtained from the Pediatric Leukemia Avatar Program within the Cancer & Blood Diseases Institute (CBDI) at CCHMC, and collected under CCHMC IRB 2010-0789. Cord blood and bone marrow specimens were collected within CCHMC IRB approved protocols 2008-0899 and 2008-0887. All human specimens were de-identified and their use has been considered as non human research (CCHMC IRB 07-07-03).
Recruitment	Recruitment was approved within the IRB approved protocols indicated above. The only selection applied was the selection of BCR-ABL+, mutant BCR-ABL or Ph-like B-ALL specimens from the Pediatric Leukemia Avatar Program (see under Population

	Characteristics). No further bias or selection of specimens was performed.
Ethics oversight	CCHMC Institutional Review Board

Note that full information on the approval of the study protocol must also be provided in the manuscript.

ChIP-seq

Data deposition

☒ Confirm that both raw and final processed data have been deposited in a public database such as GEO.

☒ Confirm that you have deposited or provided access to graph files (e.g. BED files) for the called peaks.

Data access links<br>*May remain private before publication.*	DNA sequencing data can be accessed at BioProject PRJNA675836 (https://www.ncbi.nlm.nih.gov/bioproject/PRJNA675836/). RNA and CUT&RUN sequencing data can be accessed at GEO dataset GSE196378 (https://www.ncbi.nlm.nih.gov/geo/query/acc.cgi?acc=GSE196378). The analyzed processed data are presented as Extended Data.
Files in database submission	All BED files have been deposited in GEO
Genome browser session<br>(e.g. UCSC)	Heatmaps were generated by Morpheus (software.broadinstitute.org/morpheus)

Methodology

Replicates	Cells in each CUT&RUNseq samples were obtained from the pooled bone marrow (n=3) of WT and Vav3-/- chimeric mice, and processed for CUT&run sample preparation, DNA library preparation followed by next-gen sequencing. Two independent experiments were used for reproducibility.
Sequencing depth	The libraries were sequenced on Illumina sequencer NextSeq500 or NovaSeq6000 with a sequencing depth of a minimum of 20M reads per sample. Of the total reads, more than 50% reads were uniquely mapped in Bmi1 and Ring1b CUT&RUNseq, and more than 80% mapped in the case of H2AK119Ub CUT&RUNseq. Sequencing condition used were paired end 50 basess for H2AK119Ub CUT&RUNseq, and paired end 75 bases for Bmi1 and Ring1b CUT&RUNseq.
Antibodies	The details of the antibodies (manufacturer, catalog and dilution) used for CUT&RUNseq are presented in Materials and Methods. References for specificity for anti-Ring1b and anti-H2AK119monoubiquitination marks are presented in the same section. Validation on specificity for anti-Bmi1, using CRISPR/Cas9 mediated Bmi1 deletion, in B-ALL cells is also presented in Supplementary Figure 6.
Peak calling parameters	For peak calling MACS280 was run with the FDR of 0.05. Data reported include number of peaks called, mean peak size, total reads/pairs in the treatment group, reads/pairs after filtering in treatment and fraction of reads in peaks (FRIP). Reported peaks were used in the differential binding analysis and description for specific loci, in sequence, the integer score of each peak, the fold-change at peak summit and the statistical analysis (FDR) presented as -log10q value at the peak summit. Differentially Bmi1, Ring1b and H2AK119Ub bound sites between WT and Vav3 deficient leukemic B-cell progenitors (n=2/group) were identified by using Diffbind (Differential Binding Analysis of ChIP-Seq Peak Data - 1.0.081 pipeline attached to SciDAP platform), using "Deseq2" analyses method. Only significant differentially bound sites with p-value ≤ 0.05 and with a minimum of 2 fold change (Log2Fold Change ≥ 1 and ≤ -1) were reported. Based on this, all differential peaks were divided into two groups: 1) Log2FC ≥ 1, 2) Log2FC ≤ -1. Each peak group was cleaned from duplicates based on the peak start and end coordinates and centered by peak center. Re-centered peaks were used for generating tag-density plots within 20kb radius from peak center with "Homer". For gene TSS-centered tag density plots, each peak was assigned to the nearest gene within 20kb radius from TSS. The resulted two groups of genes were de-duplicated and intersected based on the gene names thus obtaining three groups of genes. Genes from every group were recentered on the TSS and used for generating tag-density heatmap within 20kb radius from gene TSS with Homer. The tag density maps were generated using https://software.broadinstitute.org/morpheus/. The representative genome browser map of specific loci was obtained from IGV browser in "SciDAP" platform. The Venn diagram of genes with differential Bmi1, Ring1b and H2AK119Ub binding between WT and Vav3 deficient leukemic B-cell progenitors were generated using online tool "Multiple List comparator" (https://www.molbiotools.com/listcompare.php). The significance of overlapped genes and exact test of multi-set intersection were evaluated using tool (https://cran.r-project.org/), as described previously. The genes differentially bound in Vav3 deficient leukemic B-cell progenitors in comparison to their WT counterparts were subjected to gene ontology analyses (molecular and biological functions and pathway analyses) using ToppGene Suite (https://toppgene.cchmc.org/enrichment.jsp).
Data quality	As described above
Software	SciDAP (https://scidap.com, Datirium, LLC). Database for Annotation, Visualization and Integrated Discovery, v6.8

Flow Cytometry

Plots

Confirm that:

☒ The axis labels state the marker and fluorochrome used (e.g. CD4-FITC).

☒ The axis scales are clearly visible. Include numbers along axes only for bottom left plot of group (a 'group' is an analysis of identical markers).

☒ All plots are contour plots with outliers or pseudocolor plots.

☒ A numerical value for number of cells or percentage (with statistics) is provided.

Methodology

Sample preparation	The single cell suspension of leukemic mouse BM cells from hind limbs and pelvis were isolated. EYFP+ leukemia (p190-BCR-ABL+) cells from WT and Vav3-/- leukemic chimeric mouse BM were stained, identified and sorted and/or analyzed using APC-Cy7-anti-mouse CD45, PE-Cy7-anti-mouse CD19, PE-anti-mouse CD43, APC-anti-mouse IgM (all from BD Pharmingen). In the case of Bmi1 and Phlpp2 lentiviral vector transduction (EGFP+), double EYFP+ and EGFP+ transduced B-cell progenitors were sorted and/or analyzed.
Instrument	BD FACSAria II for sample sorting. BD FACSCanto devices were used for analyses.
Software	BD FACS DiVa v9.0
Cell population abundance	Populations analyzed and presented were always present at >0.1%, with a minimum of 250 events in rare event gates. Purity of the post-sort fractions was found to be > 90% in validation experiments.
Gating strategy	We applied FSC/SSC alive gate to exclude cell debris and doublets. For specific fluorochromes, the positive and negative cell populations (clusters) were demarcated at 10e3 on the axis. The dot plots or contour plots (with percentage of cell populations) were presented wherever required.

☒ Tick this box to confirm that a figure exemplifying the gating strategy is provided in the Supplementary Information.
